# Supplementary material for: Coaching doctors to improve ethical decision-making in adult hospitalised patients potentially receiving excessive treatment: Study protocol for a stepped wedge cluster randomised controlled trial
Source: PLoS One. 2023 Mar 21;18(3):e0281447. doi: 10.1371/journal.pone.0281447 (PMC10030010; doi:10.1371/journal.pone.0281447)
Supplement: S2 Appendix — (PDF) [file pone.0281447.s002.pdf]

Appendix : Coaching protocol

The intervention that is central to this study consists of implementing coaching sessions with medical doctors. These sessions aim at facilitating self-reflective and empowering leadership, and at improving medical doctors' competence in managing team dynamics with regard to ethically sensitive medical topics, in line with the theoretical framework elaborated by Van den Bulcke et al. This framework starts from the point of view that doctors need first to collect detailed information to be able to take in all conscious a decision for the benefit of the patient. Although, this seems obvious, previous studies suggest that doctors (unconsciously) prefer to remain prognostically uncertain rather than to gather the information that is required to reduce uncertainty and to effectively take decisions in the team (Palda 2005, Piers 2014). This information may be *objective* or *subjective*, and may come *directly* or *indirectly* from the patient, relatives, clinicians or any other party. All upfront clinicians may and will have potential important information about the patient, regardless of their role, knowledge or experience (Curtis 2010, Van den Bulcke 2018, ,Moynihan 2021, Michalsen 2019). To obtain all that information, the doctor in charge of the patient need to empower clinicians to speak up while guarantying a safe environment. This enables to shift from pure knowledge and experience driven discussions often led by the doctor solely, to participative knowledge, experience and value driven reflection in team (Van den Bulcke 2018). Apart from of reducing avoidance and tension in teams, and indecision in doctors, sharing emotions and values stimulates a sense of meaning, and thus ethical awareness and well-being in the team. This collective awareness enriches the ethical decision-making process for the benefit of the patient and supports the doctor to effectively communicate decisions on patient care (Van den Bulcke 2018).

Each time that at least two clinicians of the team for which a doctor is responsible indicated that

for one the patients under treatment they were facing an ethical dilemma, the doctor is invited to have a coaching session. By stimulating open speech and reflection on ethically sensitive medical topics, like disagreements on interventions, difficulties in taking EOL decisions, or challenges of dealing with conflicting opinions in the doctor-patient relationship, the intervention intended at strengthening doctors' capacities to reflect and communicate about difficult decisions in uncertain circumstances and to guide their multidisciplinary teams in dealing with such topics.

Generally speaking, coaching is a form of "leadership development where a leader has a series of contracted and confidential conversations with a coaching psychologist or development expert. It is a form of organizational learning through one-to-one conversations, which facilitates development for an individual" (de Haan, Molyn & Nilsson, 2020, p. 2). The input coachees typically bring to coaching session can cover a spectrum of themes varying between personal dynamics influencing or disturbing work situations, dilemma's and difficulties related to their work setting or exploring opportunities for growth. During coaching "coachee and coach collaborate to assess and understand the coachee and his or her leadership developmental tasks, to challenge current constraints while exploring new possibilities, and to ensure accountability and support for reaching goals and sustaining development" (Ting & Hart, 2004, p. 116). It is supporting someone to adapt his/her relationship to a specific reality. Often an individual cannot overcome the issue by him-/herself as some variables are unknown and require multiple learning loops to understand specific internal and external dynamics which inhibit our performance (Heifetz, 1997). Since furthermore, "because of the personalized nature, the high confidentiality, and the possibility for deep understanding and challenge, coachings seems to work at relational and personal depths" (de Haan, Gray & Boneywell, 2019, p. 586). Consequently, we argued that coaching could support the aim of the study.

Study Focus of the coaching

In this study coaching is implemented with a clear focus: helping medical doctors to address ethically sensitive issues in multidisciplinary teams and to optimize their decision process and leadership for the team. In hospital teams doctors typically occupy a leadership role. It is under their responsibility that crucial decisions are taken and that team dynamics take shape. Our intervention aimed at supporting them in effectuating an empowering leadership style. As Kets de Vries (Kets de Vries, 2014) indicates, "Empowering concerns the leader's ability to delegate authority to others. An empowering leader involves others in the decision-making process thereby indicating his or her high expectations and confidence in them. An empowering leader also works to minimise secrecy and to create an open and transparent environment. He or she also tolerates mistakes and failures as part of the learning process."

A key psychodynamic hypothesis guiding the intervention is that ethically sensitive medical topics are inherently anxiety provoking. They confront professionals with death and suffering, with uncertainty and powerlessness in aversive situations, and with the prospect that decisions and actions might have an aggravating impact on aversive situations. Such confrontations have a shocking effect and provoke mixed feelings. Shame, pity, compassion and guilt might come to the fore, but the bottom-line reaction to death and decay is desperate anxiety.

In her seminal psychodynamic studies on how medical professionals work, Menzies Lyth (1988) described that if this elementary anxiety is not faced and worked through, all kinds of dysfunctional defenses ruin collaborations and undermine thoughtful medical ethical decision-making. Working through means that challenging situations are faced and discussed in plain but

respectful terms, such that the complexity of the situation is acknowledged and affective reactions are contained in the interactions between professionals, patients and families. Dysfunctional defensive reactions in their turn come to the fore as people avoid thoughtful deliberation about sensitive medical topics, and shy away from such situations, e.g. by avoiding close commitment to patients, fleeing in depersonalized and neutral interactions, focusing on technical details in treatments only, emotional unresponsiveness, or scheduling no time to address worries and concerns in team meetings.

Given this challenge, the coaching sessions aim at helping medical doctors to work through ethically sensitive medical topics and facilitate a culture of open speech in their teams, defined within the context of empowering leadership. We do so by helping them to: discern which sensitive themes were currently affecting their team; reflect on how the team is currently facing or avoiding difficult questions and discussions concerning these topics; evaluate their own role and strategies in facilitating or obstructing open reflection; reflect on how they and the team are in dealing with the emotional distress that ethically sensitive medical topics.

In 2018 Van den Bulcke and colleagues reviewed literature on medical ethical decision-making and based on psychometric research of a newly constructed questionnaire (EDMCQ) they discern seven key domains that make up a medical ethical decision-making climate: (1) self-reflective and empowering leadership by physicians; (2) practice and culture of open interdisciplinary reflection; (3) culture of not avoiding end-of-life decisions; (4) culture of mutual respect in the interdisciplinary team; (5) active involvement of nurses in end-of-life care and decision-making; (6) active decision-making by physicians; and (7) practice and culture of ethical awareness. In this study, we focus on coaching doctors in self-reflective and empowering leadership, which by itself will have an effect on all other factors in the team (see Fig 2 in the protocol).

Coaching method

The coaching we implemented started from the principles described by Kets de Vries (2006, 2007) and de Haan (2008, 2014, 2019). The sessions focused on discussing: (a) challenges and opportunities in dealing with ethical and medical dilemmas; (b) challenges and opportunities in relating with colleagues and team, and with patients and families; (c) challenges and opportunities in taking up a empowering leadership role. The coach aimed at increasing awareness and efficacy in dealing with these topics, and transferring these into leadership behavior, starting from a collaborative relationship with the coachee.

Kets de Vries' coaching methodology is based on the combination of two concepts: a psychodynamic perspective and a systemic perspective. This is defined as the 'clinical paradigm', a conceptual framework that builds on psychoanalytical concepts and techniques and which takes into account the dynamics of organizational behavior (Kets de Vries, 2006). He argues that leadership behavior is driven by the interplay between conscious and unconscious processes, the so-called 'inner theatre' – the roles we have developed over the course of life and which permanently influence our thinking. Therefore this coaching methodology intends to help the leader make sense of the invisible deeper thoughts, feelings, motives and anxieties that influence his/her cognitive processes such as decision-making and daily leadership behavior.
Next to intrapersonal dynamics, the leader is also influenced by interpersonal dynamics. A leader takes up distinct formal and informal roles in various social constructions that affect his/her intrapsychic life. This implies that decision-making behavior is undoubtedly influenced by the interpretation of these interactions. Finally, leaders also act according to organizational expectations, often derived from formal rules and so-called 'organizational myths', i.e. normative

narratives that indicate how people should collaborate. As Kets de Vries (2006, p. 308) indicates, “subjection to these myths may come at the cost of personal responsibility and independence”.

It is the primary task of the coach to help leaders to explore their unconscious and invisible psychodynamic processes and structures within the context of their organization’s internal and social dynamics (Kets de Vries, 2007). “A well-trained developmental coach should at least be able to develop hypotheses and interventions both at an individual level as well as a group level” (Compernolle T., in Kets de Vries, M., Korotov, K., Florent-Treacy, E., 2007, p.35).

De Haan’s coaching methodology is based on a relational model, in which the working alliance between coach and coachee is seen as a predictor of present and future leadership effectiveness (de Haan, Gray & Boneywell, 2019, p. 585). De Haan describes relational coaching as a dual active effort. On the one hand the coach is requested to understand all leadership dynamics from the perspective of the relationships the coachee is involved in, being previous, current and in-the-moment relationships. On the other hand the coach helps the coachee to make his/her professional relationships as strong and productive as possible – considered from the perspective of the coachee. Consequently, the coach needs “to explore regularly with the coachee how the relationship is progressing” (de Haan, 2008, p.53).

Research shows that “professional coaches who specialize in the coaching profession perceive more typical nondirective and client-centered coaching behaviors over time”. It is argued that coaches “have a wide array of behavioral responses at their disposal and as they mature, they will reflect on which of their interventions to use” (de Haan & Nilsson, 2017, p. 328). This might imply that starting from a client – centred approach requires the coach to apply a wider portfolio

of potential responses and methods. By becoming more skillful over time, this will provide greater flexibility to design approaches specific to the individual (Cox, Bachkirova, Clutterbuck, 2010, p.419-420). However, this also means that the coach will always influence the conversation, both intentionally and unintentionally, by every contribution to that conversation. The coachee, as second element in the relationship, will equally bring a plethora of variables into the conversation. “An orderly, well-controlled conversation is out of the question; there are simply too many variables” (de Haan & Burger, 2014, p. 15). Consequently, de Haan argues it is the coach’s responsibility to keep a window onto the coachee’s contribution to the conversation because the coachee’s issues are data and material to work with. And it is the reciprocal relation, where coach and coachee explore the map of experiences in the moment, that defines the foundation of the coaching work.

de Haan comes with an intervention model that gives the coach a framework to conduct the conversation, based on the assumption that the coach can make the intervention from two different perspectives: the direction of the contribution (exploring or suggesting) and the nature of the contribution (supporting or confronting). In the direction – perspective the coach can decide to rather lead vs. follow the coachee. In this perspective it is a matter of either constraining the coachee’s thoughts, or following and liberating the coachee’s thoughts. In the nature of the contribution – perspective the coach can choose between challenging and supporting the coachee. In this perspective it is a matter of either building on and reinforcing the coachee’s strengths or bringing up the weaknesses and help overcoming them. (de Haan & Burger, 2014)

de Haan also contributes to the instrumentation of the coach by suggesting 4 distinct and interchangeable approaches. The first approach is person – focused, which is based on the

counselling methods as developed by Carl Rogers. The coach is observing and supporting the coachee from the his/her perspective, "attempts to shift the coachee's attention inwards and is available primarily as an accepting and attentive listener" (de Haan, 2008, p. 15). The second approach is insight-focused, which is based on the tradition of psychodynamic coaching, as described by Kets de Vries. The coach is considering the coachee from an independent perspective and "attempts, with the coachee, to understand the issue from the inside" (de Haan, 2008, p. 15). The third approach is problem-focused, which is helping the coachee with an approach to solve a problem and consequently more directive in nature. "The coach attempts to improve the situation from the outside" (de Haan, 2008, p. 16). The fourth approach is solution-focused and is a particular form of directive coaching. The coach "attempts to convert problems into positive plans and solutions" and conducts a conversation "in which coach and coachee predominantly look to the future and consider times when the problem does not arise (de Haan, 2008, p. 16).

Summarised, de Haan argues in his model of relational coaching that there should be "no restriction on the specific interventions of the coach, either in terms of nature of the contributions or in terms of the order of contributions" (de Haan, 2008, p.54). This means the coach is holding the space for the coachee and can apply a variety of relational interventions depending on coachee's reality within the moment. Consequently, there are only 3 limiting conditions for the coach: the interventions should fit in with the framework's assumptions concerning learning, development and changes; they should be focused on supporting and reinforcing the relationship and thus the coachee's learning and development process; and they should be in line with relevant ethical codes, which e.g. implies that the coach is sufficiently open to supervision (de Haan, 2008).

Coaching sessions

Each coaching session consists of an in-depth discussion of the decision making process around the case of the patient of which an alert was given.

The coach invites the coachee to describe and discuss:

- The currently challenging situation
- Perceived subjective reactions by him-/herself and others (patient, family, colleagues) to the situation
- Perceived challenges in the mutual communication about the situation, whereby attention is paid to characteristics of the own communication style and the style of the others involved
- Perceived challenges in the medical ethical decision process in the challenging situation, whereby attention is paid to differences and similarities in goals and values between all parties involved

By discussing these topics the coach aims at addressing and fostering insight in:

- Avoidance reactions in how the coached doctor, his team, and the patient and his family cope with the challenging situation
- Helping and obstructing relational patterns in relation to the team as well as in relation to the patient and his family
- Own communicative style

- Own decision making style
- Own strengths and weaknesses at the level of empowering leadership

Depending on the challenging situation and the coachee' style and trajectory the coach will direct sessions towards:

- Acknowledging and gaining insight in avoidance reactions, relational patterns, communicative processes, decision making processes and empowering leadership
- Confronting the coachee with processes he/she fails to acknowledge
- Reflecting with the coachee on dealing with and intervening upon such processes

Coaching intervention

Four coaching interventions of 4 months were conducted in 10 internal medicine and neurology wards over a period of 16 months. During that period daily individual coaching took place in self-reflection and self-regulation with regard to ethical decision-making in patients potentially receiving excessive care and in coping with group dynamics in the interdisciplinary team, with the intention to achieve following specific objectives:

a) Learning to acknowledge the patient's (and relatives') subjective goals, emotions and values, and separate them from own and colleagues' subjective goals, emotions and values trigged by that situation

b) Learning to acknowledge patient's (and relatives'), colleagues' and own spontaneous defensive avoidance strategies in coping with difficult and aversive care-related situations, like end-of-life decisions.
c) Learning to identify and separate internal avoidance strategies from external barriers to better delignate the responsibilities of each stakeholder in the process
d) Learning to cope more effectively with these internal and avoidance strategies and external barriers to enable more appropriate and timely decisions for the benefit of the patient.
e) Learning to integrate newly acquired insights into an adapted way of thinking and relating with others to establish a sustainable effect with regards to ethical decision-making.
f) Learning to transfer these insights into empowering leadership behaviour which contributes to dialogue during the interdisciplinary meeting

Success indicators

1. The intervention was based on the procedural alert. If not, please specify the trigger.
2. The doctor is aware of his/her subjective reaction to the uncertainty of the prognosis.
3. The doctor is aware of his/her subjective reaction to the anticipated decision.
4. The doctor shared his understanding about the doubt of others in relation to the described therapy.
5. The doctor gave proof through specific comments of insight into the perspective of the patient.

6. The doctor gave proof through specific comments of insight into the perspective of the family.
7. The doctor gave proof through specific comments being able to separate internal avoidance strategies from external barriers
8. The doctor described his fear for conflict.
9. The intervention took place in an appropriate coaching setting (i.e. confidential, discrete, serene, not be pressured by time,…).
10. We were able to process multiple learning loops which fostered the physician's thinking.
11. The doctor demonstrates his ability to implement newly acquired insights during the interdisciplinary meeting.
12. The doctor applies empowering leadership skills during the interdisciplinary meeting.

Coaching conversation

As referred, multiple learning loops might be needed to understand specific internal and external dynamics which inhibit our performance (Heifetz, 1997). In order to give doctors the possibility to achieve this objective, each coaching conversation also followed a specific workflow with multiple learning loops to foster the reflection process within the moment (Killburg, 2000).

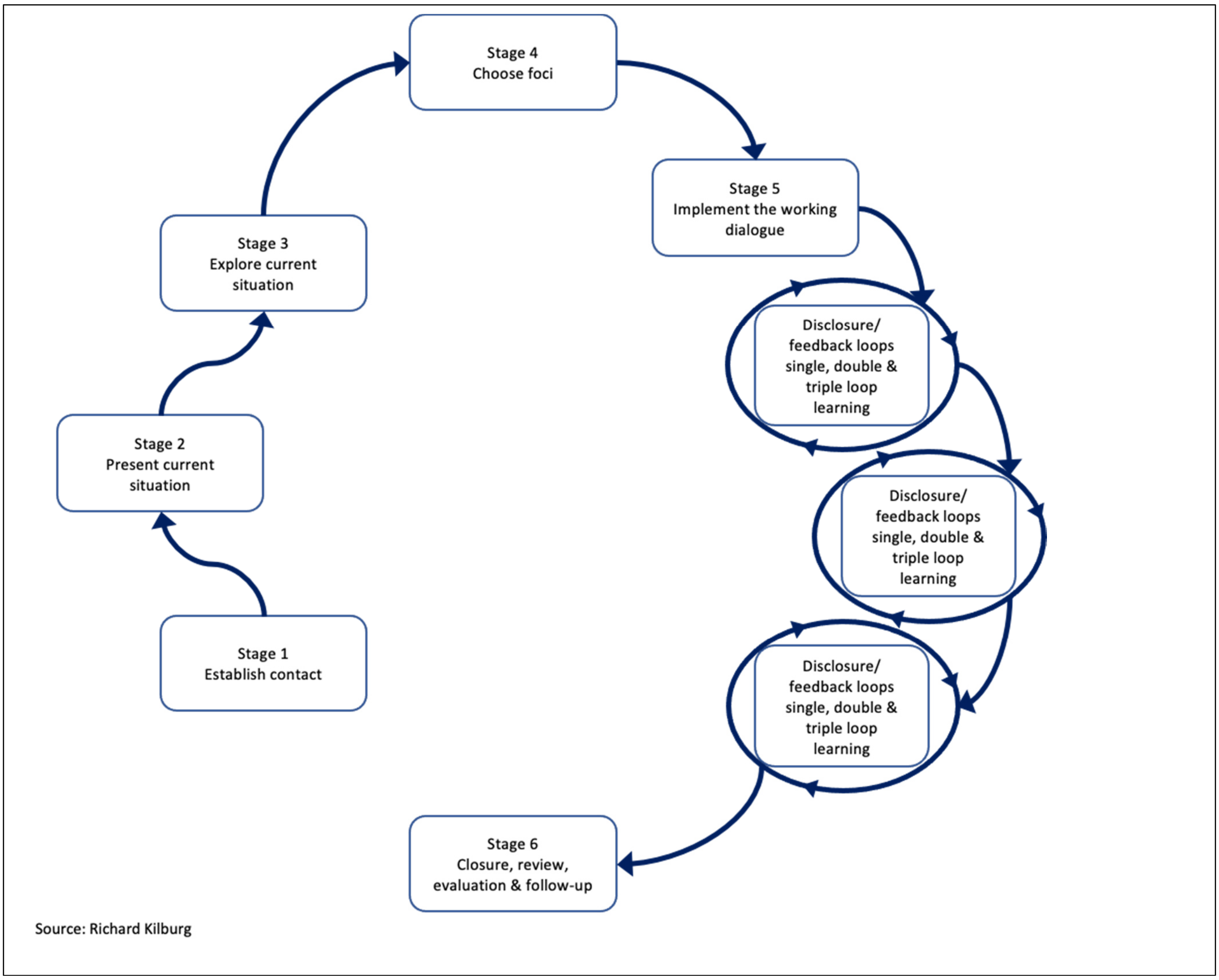

Quality assurance

To secure the quality of the intervention the coach worked under the guidelines of the Ashridge Code of Conduct (see appendix).

The quality of the coaching we implemented was also supervised by an independed third-party supervisor; governed by ethical standards and principles as adopted from de Haan (de Haan, 2019). Five moral foundations which support integrity in coaching and research were applied:

independence to guard the physician's autonomy, informed consent so the physician knows what he/she is getting involved in, confidentiality to safeguard the physician, respect and diversity to allow multiple voices and perspectives and care about vulnerable parties, integrity and trust to comply with legislation and to handle data in an ethical way.

This supervisor had is normative, formative and restoring role (Proctor, 2006). The normative role of the supervision aimed at monitoring the quality of the coaching methodology and ethical aspects with regard to the study aims. The formative role aimed at supporting the coach in further developing and refining his/her skills in general and more specifically with regard to the study aims. The restorative role aimed at guarantying the energy of the coach and at resolving potential conflicts due to emotional or unconscious dynamics in the team.

Coaching Supervision is about ensuring high quality coaching provision and takes the form of ongoing meetings between the supervisor and the coach. One way of looking at the process of supervision is provided by the Seven-eyed model (Hawkins and Shohet, 2000). Originally developed for use with psychotherapists and counsellors, it is now being applied to coaching and mentoring. It describes the 7 areas that supervision can focus on:

1. **The physician**: The focus is on the physician's situation – the case the physician wants help with, how they present the issues and the choices they are making.
2. **The coach's Interventions**: The focus is on the interventions the coach made, how and why they made them, and what else they might have done.

3. **The relationship between the coach and physician**: The focus is on neither the coach nor the physician but on the conscious and unconscious interactions between the two of them, so that the coach develops a better understanding of the dynamics of the coaching relationship.
4. **The coach**: The focus is on the coach's own experience as an instrument for registering what is happening beneath the surface of the physician's system.
5. **The supervisory relationship**: The focus is on what the coach has absorbed from the physician's system and how it may be playing out in the relationship between coach and supervisor.
6. **The supervisor's self-reflection**: The focus is the supervisor's "here and now" experience with the coach and how this can be used to shed light on the coach/ physician relationship.
7. **The wider context**: The focus is on the wider organisational, social, cultural, ethical, and contractual context within which the supervision is taking place.

In focusing on areas 1-3, the supervision is concerned with reflecting on the coaching session itself – its content, the interventions made, and the dynamics of the coaching relationship.

In areas 4-6, the supervision is concerned with the coaching session as it is reflected in the here and now experience of the supervision session.

The value of this model is that it maps the areas that supervision can focus on, making it easier to ensure that we have covered the ground.

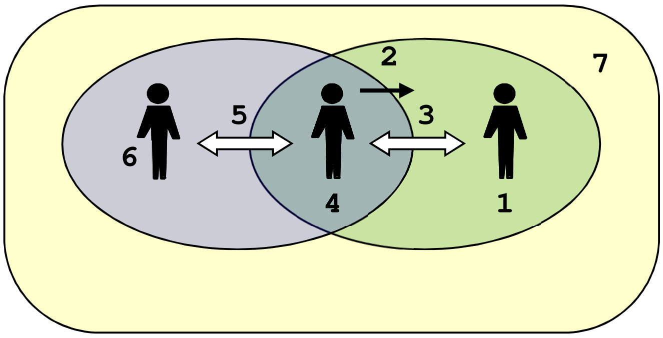
2
7
5
3
6
4
1

Bibliography

Curtis JR, Vincent JL. Ethics and end-of-life care for adults in the intensive care unit. *Lancet* 2010;376(9749), 1347–1353. https://doi.org/10.1016/S0140-6736(10)60143-2

Cox, E., Bachkirova, T., Clutterbuck, D., (2010). *Handbook of coaching*. London: Sage

de Haan, E. (2008). *Relational Coaching*. West Sussex: Wiley

de Haan, E. Burger, Y., (2014). *Coaching with colleagues.* Basingstoke: Palgrave MacMillan

de Haan, E. (2019). *Critical moments in executive coaching.* New York: Routledge

de Haan, E., Gray, D.E. & Bonneywell, S. (2019). Executive coaching outcome research in a field setting: A near-randomized controlled trial study in a global healthcare corporation. Academy of Management Learning and Education, 18.4, 1- 25. https://doi.org/10.5465/amle.2018.01588

de Haan, Large-scale survey of trust and safety in coaching supervision: Some evidence that we are doing it right. *International Coaching Psychology Review* l *Vol. 12 No. 1 March 2017*

de Haan,E., Nilsson, V. O. (2017). Evaluating coaching behavior in managers, consultants, and coaches: a model, questionnaire and initial findings. Consulting Psychology Journal: Practice and Research, 69.4, 315-333.

de Haan, E., Molyn, J., & Nilsson, V. O. (2020). New findings on the effectiveness of the coaching relationship: Time to think differently about active ingredients? *Consulting Psychology Journal: Practice and Research.* Advance online publication. https://doi.org/10.1037/cpb0000175

Hawkins, P., Shohet, R. (2012). *Supervision in the helping professions.* Maidenhead: McGraw-Hill Education

Kets de Vries, M. (2006). *The leader on the couch.* West Sussex: Wiley

Kets de Vries, M., Korotov, K., Florent-Treacy, E. (2007). *Coach and couch.* New York: Palgrave Macmillan

Kets de Vries, M. (2014). *Global Executive Leadership Mirror; facilitator guide*. London: KDVI

Kilburg, R. (2000). *Executive Coaching*. Washington: American Psychological Association

Michalsen A, Long AC, DeKeyser Ganz F, et al. Interprofessional Shared Decision-Making in the ICU: A Systematic Review and Recommendations From an Expert Panel. *Crit Care Med* 2019;47(9), 1258–1266. https://doi.org/10.1097/CCM.0000000000003870

Moynihan KM, Taylor L, Crowe L, et al. Ethical climate in contemporary paediatric intensive care. *J Med Ethics* 2021;medethics-2020-106818. Advance online publication. https://doi.org/10.1136/medethics-2020-106818

Palda VA, Bowman KW, McLean RF, et al. "Futile" care: do we provide it? Why? A semistructured, Canada-wide survey of intensive care unit doctors and nurses. *J Crit Care* 2005;20(3), 207–213. https://doi.org/10.1016/j.jcrc.2005.05.006

Piers RD, Azoulay E, Ricou B, et al. Inappropriate care in European ICUs: confronting views from nurses and junior and senior physicians. *Chest*, 2014;146(2), 267–275. https://doi.org/10.1378/chest.14-0256

Proctor, B. (2006). Contracting in supervision. In C. Sills (Ed.). *Contracts in counselling & psychotherapy* (pp. 161-174). London: Sage Publications Ltd.

S. Ting, E.W. Hart (2004). Formal coaching. In: C.D. McCauley, E. Van Velsor (Eds.), The Center for Creative Leadership Handbook of Leadership Development, John Wiley & Sons, San Francisco (2004), pp. 116-150

Van den Bulcke et al. (2018). Ethical decision-making climate in the ICU: theoretical framework and validation of a self-assessment tool. *BMJ Qual Saf, 27(10),*781–789
