# Supplementary material for: Coaching doctors to improve ethical decision-making in adult hospitalised patients potentially receiving excessive treatment: Study protocol for a stepped wedge cluster randomised controlled trial
Source: PLoS One. 2023 Mar 21;18(3):e0281447. doi: 10.1371/journal.pone.0281447 (PMC10030010; doi:10.1371/journal.pone.0281447)
Supplement: S1 Protocol — (PDF) [file pone.0281447.s003.pdf]

Coaching doctors in ethical decision-making : a stepped wedge cluster randomized trial in 10 departments of the Ghent University Hospital.

Acronym / Protocol code	CODE-study
Protocol version and date	Version 2.0 November 19, 2021
Phase	NA
EudraCT n°	NA
Sponsor	Ghent University Hospital
Financial/Material Support:	Fund for Innovation and Clinical studies of the Ghent University Hospital / FWO 1800518N

Coordinating Investigator:

Dominique D. BENOIT

Department of Intensive Care Medicine

Ghent University Hospital

Coaching doctors in ethical decision-making : a stepped wedge cluster randomized trial in 10 departments of the Ghent University Hospital

Protocol Coordinating Investigator signature page

I certify that I will conduct the study in compliance with the protocol, any amendments, GCP and the declaration of Helsinki, and all applicable regulatory requirements.

Investigator:

Name: Dominique D. BENOIT

Function: MD, PhD, Head of the Department of Intensive Care Medicine

Institution: Ghent University Hospital, Ghent, Belgium

Date: November 19 2021 **Signature:**

TABLE OF CONTENTS

LIST OF ABBREVIATIONS 6

1. Protocol Summary 7
   - 1.1. Title 7
   - 1.2. Protocol specifics 7
   - 1.3. Study Type and Study Phase 7
   - 1.4. Aim of the study (including primary endpoints) 7
   - 1.5. Subjects 7
     - 1.5.1. Number of subjects 8
     - 1.5.2. Target group 8
   - 1.6. Inclusion and exclusion criteria 8
   - 1.7. Study Interventions 8
     - 1.7.1. Schematic overview of the data collection & interventions 10
   - 1.8. Study duration 11
     - 1.8.1. For an individual subject 11
     - 1.8.2. For the whole study 11
2. Rationale and background 12
   - 2.1. Rationale 12
   - 2.2. Background 14
   - 2.3. Risk/Benefit Assessment 16
   - 2.4. Limitations 17
3. Objectives 18
   - 3.1. Primary Objectives 18
   - 3.2. Secondary Objectives 19
   - 3.3. Tertiary Objectives 20
4. End Points + Time Points 21
   - 4.1. Primary End Points + Time Points 21
   - 4.2. Secondary End Points + Time Points 21
   - 4.3. Tertiary/Exploratory End Points 22
   - In order to evaluate the quality of the implementation of coaching, we added process measures based on the RE-AIM implementation framework. 22
5. Study design 23
   - 5.1. Description of study design 23
     - 5.1.1. For an individual subject 23

5.1.2. For the whole study 23
5.2. Estimated duration of the study 23
5.2.1. For an individual subject 23
5.2.2. For the whole study 24
6. Inclusion and Exclusion Criteria 24
6.1. Inclusion Criteria 24
6.2. Exclusion Criteria 24
6.2.1. Screen failures 24
7. Target Population 25
7.1. Subjects 25
7.1.1. Number of subjects and planned recruitment rate 25
7.1.2. Withdrawal and replacement of subjects 25
7.2. Method of recruitment 25
7.3. Screening 26
8. Study Specific Procedures 28
8.1. Randomisation/blinding 28
8.2. Randomisation/blinding 28
8.3. Overview of collected data 29
8.4. Schematic overview of the data collection & interventions 31
9. Statistical Considerations 32
9.1. Sample size calculation 32
9.2. Type of statistical methods 33
9.3. Statistical analysis team 33
9.4. Interim analysis 33
10. Data handling 34
10.1. Method of data collection 34
10.1.1. Case Report Form 34
10.2. Data storage 34
10.3. Archiving of data 34
10.4. Access to data 35
11. Monitoring/Auditing/Inspection 36
11.1. Monitoring 36
11.2. Inspection 36
11.3. Protocol Deviation policy 36
11.4. Serious breach to GCP and/or the protocol 36
12. Ethical and legal aspects 37

12.1. Good Clinical Practice 37
12.2. Informed Consent 37
12.3. Approval of the study protocol 38
12.3.1. General 38
12.3.2. Protocol amendments 38
12.4. Confidentiality and Data Protection 38
12.5. Liability and Insurance 39
12.6. End of Study Notification 39
13. Publication policy 40
14. Reference List 40
15. Appendices 49
15.1. Appendix 1: Coaching protocol 49

LIST OF ABBREVIATIONS

APPROPRICUS	=	Appropriateness of care in the ICU
CI	=	Coordinating Investigator
CT	=	Clinical Trial Unit
DISPROPRICUS	=	Disproportionate care in the ICUs
DNIR	=	Do-not-intubate and -resuscitate
EC	=	Ethics Committee
EDMCQ	=	Ethical Decision-making Climate Questionnaire
eCRF	=	electronic Case Report Form
EDC	=	Electronic Data Capture
EPD	=	Electronic Patient Dossier
FPI	=	First Patient In
GCP	=	Good Clinical Practice
GDPR	=	General Data Protection Regulation
HIRUZ	=	Health, Innovation and Research Institute UZ Ghent
IB	=	Investigator's Brochure
ICF	=	Informed Consent Form
ICU	=	Intensive Care Unit
LVLS	=	Last Visit, Last Subject
PI	=	Principal Investigator

1. Protocol Summary

1.1. Title

Coaching doctors in ethical decision-making : a stepped wedge cluster randomized trial in 10 departments of the Ghent University Hospital

1.2. Protocol specifics

EudraCT number : NA

Sponsor : Ghent University Hospital

1.3. Study Type and Study Phase

Stepped wedge cluster randomized study

1.4. Aim of the study (including primary endpoints)

The aim of this study is to investigate whether coaching doctors in self-reflective and empowering leadership, and in the management of team dynamics with regard to hospitalized patients potentially receiving excessive treatment during 4 months 1) improves ethical decision-making (primary objective) and 2) reduces the burden on patients, relatives, clinicians and the society (secondary objective). The improvement in quality of ethical decision-making will be assessed *objectively* via the incidence of written do-not-intubate and -resuscitate (DNIR) orders (first primary endpoint) in adult patients potentially receiving excessive treatment during their first hospitalization and *subjectively* via the ethical decision-making climate questionnaire (EDMCQ) [1] that will be filled out by the team (second primary endpoint) at the start and end of the twelve months study period.

1.5. Subjects

- Junior and senior doctors (for the intervention)
- Nurses, junior and senior doctors (for rating the EDMCQ and giving perceptions when a patient potentially receiving excessive treatment)
- Head nurses and medical head of department (for department characteristics)
- Patients and their relatives (for rating quality of care / communication and psychosocial well-being)

1.5.1. Number of subjects

In total 50 à 75 junior doctors, 50 à 75 senior doctors (including medical head of department) and 500 à 600 nurses (including head nurses) working in 10 departments of the Ghent University Hospital are eligible for this study and about 1400 to 1700 patients potentially receiving excessive treatment together with one of their relatives. The participating departments are : Cardiology, Gastro-enterology and Hepatology, General Internal Medicine, Geriatrics, Hematology, Medical Oncology, Neurology, Nephrology, Pulmonology and the Medical ICU.

1.5.2. Target group

All junior and senior doctors taking care of hospitalized patients in the 10 participating departments of the Ghent University Hospital are eligible for the intervention. They will be coached with regard to hospitalized patients who are perceived as receiving excessive treatment according to two or more different clinicians (nurses, junior doctors or senior doctors) in the team. These patients will subsequently be included for data collection at the patient, relative and societal level. **"Patients who are perceived as receiving excessive treatment according to two or more clinicians" will be denominated from now on as *"patients potentially receiving excessive treatment"* throughout the entire protocol for ease of reading.**

1.6. Inclusion and exclusion criteria

- junior and senior doctors
  - inclusion: all junior and senior doctors ((including medical head of department) taking care of hospitalized patients in the 10 participating departments of the Ghent University Hospital
- nurses
  - inclusion: all nurses (including the head nurses) taking care of hospitalized patients in the 10 participating departments of the Ghent University Hospital
- patients and family members
  - inclusion: first hospitalization of patients who are potentially receiving excessive treatment in the 10 participating departments of the Ghent University Hospital
  - exclusion:
    - being younger than 18 years old
    - not able to fill in Dutch questionnaires

1.7. Study Interventions

The intervention in junior and senior doctors consists of <u>4 components:</u>

1) *One* interactive session of half a day focusing on the concepts of medical-ethical decision-making, the psychological challenge of dealing with ethically sensitive medical topics, and

empowering leadership. This session is for junior and senior doctors and will present coaching as a support mechanism for professional development.

2) Observation of the interdisciplinary meeting as input for coaching on ethical decision-making (with focus on self-reflective and empowering leadership and on managing group dynamics)
3) Individual coaching on the spot in self-reflective and empowering leadership and in managing groups dynamics with regard to ethical decision-making about patients potentially receiving excessive treatment during the 4 months intervention period, and in absence of such patients, every item with regard to ethical decision-making that is important for the coachee. Every doctor will be invited to participate to at least 8 coaching sessions, to be extended on request, of 1 hour during the intervention period.

Focus of the coaching

a) Learning to acknowledge the patient's (and relatives') subjective goals, emotions and values, and separate them from own and colleagues' subjective goals, emotions and values trigged by that situation.
b) Learning to acknowledge patient's (and relatives'), colleagues' and own spontaneous defensive avoidance strategies in coping with difficult and aversive care-related situations, like end-of-life decisions.
c) Learning to identify and separate internal avoidance strategies from external barriers to better delignate the responsibilities of each stakeholder in the process.
d) Learning to cope more effectively with these internal and avoidance strategies and external barriers to enable more appropriate and timely decisions for the benefit of the patient.
e) Learning to integrate newly acquired insights into an adapted way of thinking and relating with others to establish a sustainable effect with regards to ethical decision-making.
f) Learning to transfer these insights into empowering leadership behavior which contributes to dialogue during the interdisciplinary meeting

Quality of the coaching

Coaching and supervision will be done by professionals who are certified by international standards, hold a degree in human sciences and have experience in leadership development. The independent experienced supervisor has a normative, formative and restoring role. The normative role of the supervision aims at monitoring the quality of the coaching methodology and ethical aspects with regard to the study aims. The formative role aims at supporting the coach in further developing and refining his/her skills in general and more specifically with regard to the study aims. The restorative role aims at guaranteeing the energy of the coach and at resolving potential conflicts due to emotional or unconscious dynamics in the clinical team. This will be done in collaboration with the "cell wellbeing" of the Ghent University Hospital, in line with the contracted mandate to securing the ethical boundaries of the supervision.

We refer to the appendix for the detailed methodology

a) the methodology, used by the coaches during the intervention
b) the quality assurance process, used during supervision
c) the references to the international standards

4) During the intervention coaches and doctors in charge will be informed of the presence of a patient potentially receiving excessive treatment in their ward by an electronic alert. Every clinician will be invited to provide daily a perception of excessive treatment via the EPD when he/she feels that the treatment that is provided to his/her patient is excessive. Excessive treatment is defined as treatment that is perceived to be no longer consistent with the expected survival or quality ("too much treatment") or that is provided against the patient's or relatives' wishes [2]. Once a patient is identified by two or more different clinicians, an email will be sent to coaches and doctor in charge of the patient.

1.7.1. Schematic overview of the data collection & interventions

a) Schematic overview of data collection

	T0	T1	T2	T3	T4
Department characteristics by head nurse and medical head of department	X			X	
Personal characteristics and EDMCQ by nurses and doctors	X			X	
Perceptions of nurses, junior, senior doctors					
Inclusion of patients identified as potentially receiving excessive treatment					
Data collection* in patients and relatives					
Data collection in medical charts					
Data collection about coaching					
Evaluation of the intervention in participants (survey and focus groups)					

T0: start of the study period of 10 to 12 months
T1: start of the intervention period (depends on the randomization per team)
T2: end of the intervention period of 4 months (depends on the randomization per team)
T3: end of the study period of 10 to 12 months
T4: post-intervention (depends on the randomization per team)

*Patients and their families are asked
- during hospitalization: informed consent and basic characteristics during hospitalization
- 3 weeks after discharge: survey on wellbeing, rating of quality of care, communication and decision-making

*Patients are asked
- 1 year after discharge: survey on living situation

b) Schematic overview of stepped wedged protocol (Fig 1)

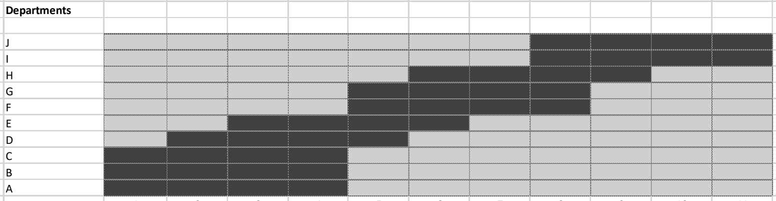

Intervention Month	1	2	3	4	5	6	7	8	9	10	11		Total
FTE individual coaching	0,8	0,8	0,8	0,8	0,8	0,8	0,8	0,8	0,8	0,8	0,8		
FTE observation team meeting	0,2	0,2	0,2	0,2	0,2	0,2	0,2	0,2	0,2	0,2	0,2		
Exp. number of patients for inclusion	140	140	140	140	140	140	140	140	140	140	140	140	1680
Exp. number of patients without DNIR	88	88	88	88	88	88	88	88	88	88	88		
Exp. number of patients on intervention departments	27	36	45	45	36	36	27	45	27	18	18		
Exp. number of coaching sessions on intervention departments	54	72	90	90	72	72	54	90	54	36	36		

Based on the results of a pilot study (Table 1), we expect that 140 patients per month will be identified by clinicians as potentially receiving excessive care, of whom 88 without a DNIR code, yielding to 9 patients and 18 coaching sessions (one junior and one senior doctor per patient) per month per department.
As 2 to 5 departments will have the intervention at the same time, the expected number of coaching sessions varies between 36 and 90 sessions per month.
The black bars indicate the intervention period of 11 months accross all 10 departments and 1 month to take holidays into account where no coaching happens.

All 10 departments will be randomly assigned to start a 4-month coaching period in month k=1,...,10 following a stratified design.
In particular, the 3 departments with the highest incidence of written DNIR orders (based on historical data, Table 1)
will be randomly assigned to start the intervention in months 2, 4 and 6 (each time together with another ward).
The 7 other wards will be randomly assigned to start the intervention according to this schematic overview inorder not to transcend 5 wards per month.

The intervention will be conducted in 10 departments over 12 months. A maximum of 6 departments will be coached at the same time resulting in a maximum expected number of coaching sessions of 90 per month, 22 per week or 5 to 6 per day for a 0.8 FTE working 4 days per week.

1.8. Study duration

1.8.1. For an individual subject

- Junior and senior doctors are coached for 4 months (T1-T2)
- Nurses, junior and senior doctors' perceptions when excessive treatment: 10 to 12 months (T0-T3)
- Head nurses and medical heads of department (for department characteristics): 10 to 12 months (T0-T3)
- Patients are surveyed 3 weeks and 1 year after hospital discharge
- Family members are surveyed 3 weeks after hospital discharge

1.8.2. For the whole study

22 months including 10 months (+ 2 months of holiday or absence of the coaches because of any reason) of coaching and the collection of the combined endpoint after one year

2. Rationale and background

2.1. Rationale

In order to improve care at the end-of-life for seriously ill patients and their families, many research groups around the world focus on advance care planning, and earlier integration of palliative care. To our knowledge, our research group is the first to focus on the value of subjective impressions (perceptions) of clinicians in addition to objective criteria to identify timely patients potentially receiving excessive care.

We chose this approach more than a decade ago [3] because of the following reasons :

1) Although the medical community put tremendous efforts in trying to improve prognostication via objective factors or scoring systems [4], their predictive value is becoming less important because medical and technical innovations frequently exclude patients' spontaneous death. Nowadays, patients die mainly after doctors have decided to withhold or withdraw treatment together with the patient or their relatives [5-7]. Moreover, prognostic criteria or scoring systems fail to predict outcome at the individual patient level [8].
2) Despite the availability of universal objective prognostic factors for many diseases, a large variability in written DNIR orders, utilization in health care resources at end-of-life, palliative care and place of death have been observed across continents, countries, hospitals, wards, doctors and patients, even after adjustment for the case-mix [9-17]. This indicates that subjective factors at the personal ("style"), team ("climate") and country ("culture") level are more important than objective factors during ethical decision-making. However, subjective factors are rarely acknowledged and expressed by clinicians, more specifically by doctors at the bedside [2,19,20].
3) Focusing tenaciously on objective criteria only, bears witness to a defensive avoidance strategy that draws attention away from difficult and aversive treatment-related situations that might provoke anxiety [19-22], like end-of-life situations, which require ethical decision-making. Nowadays, this is potentially all the more problematic because of point 1.
4) Focusing tenaciously on objective criteria puts accountability outside oneself and may therefore protect doctors from acting, whereas subjective criteria should be integrated in the decision-making process in order to take participative decisions for the benefit of the patient [1,2,19,20].
5) Balanced medical ethical decision-making and empowering leadership should take into account both objective criteria and subjective information concerning goals, emotions and values of patients and relatives [1,2,19,20], and assessments of all parties in the multidisciplinary team [1,6]. Although the four ethical principles (beneficence, non-maleficence, autonomy and distributive justice) and their critical considerations remains essential, meaning-making through human stories (narrative ethics) and shared understanding of patient's situations through dialogue (hermeneutics) [1,23,24] is today all the more important because of point 1.
6) Difficult and aversive treatment-related situations trigger professionals' subjective goals, emotions and values too, which may give rise to disagreement [1,5,6,18-21] and may result in avoiding ethical decision-making and may undermine empowering leadership. Therefore, group dynamics concerning decision-making and leadership in medical teams should be monitored and discussed explicitly.
7) Subjectivity is prone to bias. Nevertheless, it is of significant prognostic value, more specifically when expressed by several clinicians. As already mentioned above the

probability of being alive, at home with a good quality of life one year after ICU admission was only 7% in patients who were perceived as receiving excessive care by two or more clinicians in the multicentre DISPROPRICUS study [2]. Because according to the noble sense of the word, care can never be excessive or inappropriate, we decided to use the term treatment in the current project instead of care, in line with a recent expert consensus meeting [7].

Because of all these reasons, concordant perceptions of excessive treatment by two or more clinicians can be considered as an *ideal palliative care trigger,* a signal that the team should reflect about the quality care of care that is provided to the patient and whether the treatment is in balance with the medical condition of the patient and the patients' goal of care. Moreover, this approach has the advantage of selecting patients on "human grounds" rather than on abstract objective criteria. This further stimulates engagement and accountability in clinicians, more specifically in nurses and junior doctors. However, creating a climate which enables clinicians to speak up without being afraid of a verbal or non-verbal reprimand, or being considered as "incompetent" or "not respectful" towards higher ranked professionals like doctors, will therefore first be necessary [1]. The key position of doctors in the ethical decision-making process and the fact that senior doctors tend to overrate their communication, leadership- and decision-making capacities in general [25] and more specifically at end-of-life [2,26-28] naturally points to them for these interventions. This is more specifically true in units with a poor ethical climate [2,27].

We refer to Van den Bulcke et al for the detailed theoretical framework [1]. This framework starts from the point of view that doctors need first to collect detailed information to be able to take in all conscious a decision for the benefit of the patient. Although, this seems obvious, previous studies suggest that doctors (unconsciously) prefer to remain prognostically uncertain rather than to gather the information that is required to reduce uncertainty and to effectively take decisions in the team [26, 29]. This information may be *objective* or *subjective*, and may come *directly* or *indirectly* from the patient, relatives, clinicians or any other party. All upfront clinicians may and will have potential important information about the patient, regardless of their role, knowledge or experience [1, 6, 24, 30]. To obtain all that information, the doctor in charge of the patient need to empower clinicians to speak up while guarantying a safe environment. This enables to shift from pure knowledge and experience driven discussions often led by the doctor solely, to participative knowledge, experience and value driven reflection in team [1]. Apart from of reducing avoidance and tension in teams, and indecision in doctors, sharing emotions and values stimulates a sense of meaning, and thus ethical awareness and well-being in the team. This collective awareness enriches the ethical decision-making process for the benefit of the patient and supports the doctor to effectively communicate decisions on patient care [1].

Creating a safe climate which enhances inter-professional shared decision-making for the benefit of the patient requires specific self-reflective and empowering leadership skills (including the management of group dynamics in the interdisciplinary team) [1,2,30-33] which we want to develop with this intervention. These skills will also help doctors during patient and family meetings which will enable doctors to better take into account the patient's and family's wishes.

In contrast to other investigators who focused on the effect of a consultant outside the team (communication facilitator, palliative or ethics consultant…) [34-39], we preferred to perform an intervention inside the team because we believe in the personal development of junior and

senior doctors working in our hospital (relational coaching from a psychodynamic and systemic perspective, see appendix) and because most often doctors still have to trigger or give their approval for consultations of experts outside the team. We also decided to coach doctors in self-reflective and empowering leadership on the field via real repetitive patient situations because we think this will be more effective. This is in contrast with previous leadership development programs that mainly targeted resident doctors or doctors in mid-level position and focused on skills training and technical and conceptual knowledge via workshops, lecture, plenary speeches or groups discussion, respectively. Moreover, although all 45 studies report positive outcomes, few report system-level effects such as improved performance on quality indicators or customer satisfaction [40].

The intervention will be compared with usual treatment. Except from a treatment-limitation -decisions guideline which focuses on the legal and deontological framework, no other guideline with regard to ethical decision-making has been implemented at the Ghent University Hospital. Therefore, we expect a high variability in ethical decision-making across wards. Besides the primary outcomes (written DNIR orders and EDMCQ) all factors related to quality of end-of-life care (mortality and survival with a good quality of live at home, number of interdisciplinary and family meetings, number of communications with referring doctors and general practitioners, symptom management…) will be analyzed as tertiary outcomes. Since time will be taken into account in our analysis, we will also be able to assess the impact of the number of coaching sessions on these outcomes within and between wards.

2.2. Background

The main goal of medicine is to reduce morbidity and mortality and to restore health without prolonging the suffering of patients. However, over the last few decades the fast technical and medical progress poses a significant challenge to doctors, who are asked to find the right balance between life-prolonging and palliative care [1,2, 5-7]. Several large multicenter studies indicate that clinicians in Belgium less often reflect about the quality of care provided to patients with a high probability of dying (prospective study) and more often provide aggressive treatment at end-of-life (retrospective study) in comparison with clinicians in surrounding countries. In a study including patients who were 79±7 years old and died with cancer, Bekelman et al found that 51% died in an acute care hospital and 17% were admitted in the ICU in the last 180 days of life in Belgium as compared to respectively 38% and 8% in the Germany and 29% and 10% in the Netherlands [12]. De Roo et al found that the percentage of time spent in the hospital in the last month of life in cancer patients was also the highest in Belgium (25%), followed by Italy (22%), Spain (18%) and the Netherlands (14%) [41]. These figures suggest that the trend in aggressive treatment at end-of-life together with the resulting shift in place of death (from home to care homes, to acute hospitals and finally to the ICU) that has been observed in the last decades in Western countries [13, 41-44] is more pronounced in Belgium than in the surrounding countries. This evolution is in contrast with the majority of patients who prefer to die at home [44] in dignity and in presence of their loved ones [44-46]. Besides the fact that Belgium has one of the highest number of ICU beds per inhabitant in Europe [47], less openness to discuss ethical sensitive issues with patient and relatives, and between and within teams may further contribute to this finding. For instance, Meeussen et al found that end-of-life issues were less often discussed in the last month of life in cancer patients in Belgium (68%) than in the Netherlands (88%) [48]. End-of-life treatment preferences were also less often known (43% vs. 67%, respectively). Moreover, in a recent prospective study performed in 68 ICUs across 12 European countries and the United States,

Benoit el al found that the ethical decisions-making climate in Belgium and more specifically in Flanders was closer to units in Italy and Portugal than to units in Denmark, the Netherlands or France, although variability within countries was observed [2, 49]. In these units, there was less room to reflect about the quality of care and for formal and informal discussions in team, dying was more often considered as therapeutic failure and decisions in general and at end-of-life were more often postponed [2, 50]. Decision-paralysis was also objectively confirmed at the patient level in these units, hereby validating our EDCMQ instrument [2].

From the figures above, one may expect that clinicians are often confronted with patients potentially receiving excessive treatment, more specifically in Belgium and in the Ghent University Hospital. Three large multicentre studies have measured the prevalence of perception of excessive treatment in clinicians. In the APPROPRICUS study performed in 9 European countries and Israel, 27% of the ICU clinicians working on the day of study claimed to take care of at least one patient who received inappropriate, mostly excessive treatment [3]. Anstey et al found a prevalence of 38% among 1363 clinicians working in 56 Californian ICUs. In contrast to the APPROPRICUS study, these investigators found a difference in prevalence between doctors and nurses (51% vs. 36%, p<0.001) [51]. Benoit et al recently assessed the incidence of perceptions of excessive treatement by clinicians in patients admitted in 68 ICUs in Europe and the United States [2]. Of the 1761 patients admitted over a 28 day period, 369 (20.9%) and 181 (10.2%) were identified as receiving excessive treatment by at least one and by at least two clinicians, respectively. Whereas patients potentially receiving excessive care had a 7% probability of surviving at home with a good quality of life at one year, the probability of receiving a written DNIR order was 30% only, varying from 35% in units with a good vs. 20% in units with poor ethical decision-making climate (p=0.011). In order to perform the power analysis for this study, we measured the incidence of patients potentially receiving excessive treatment in the 10 wards of the Ghent University Hospital willing to participle with this study. The measurement was performed during one week in July 2019 after approval of the department heads. In total, 3379 perceptions of excessive treatment were collected in 3703 clinicians. The response rate was 91%. Of the 268 patients admitted in these wards, 32 (11.9%) were potentially receiving excessive treatment and only 12 (37.5%) had a written DNIR order (Table 1). This is completely in line with the ICU setting [2]. The incidence of patients potentially receiving excessive treatment and written DNIR order varied from 5% to 24% and from 20% to 67% in patients potentially receiving excessive care between departments, respectively. This means that the ethical principle of not harming was potentially violated in 20 patients admitted in these wards during that week given the risk of receiving cardio-pulmonary resuscitation or of being referred to the ICU in case of deterioration despite the poor excepted outcomes at one year [52,53].

Table 1 : Incidence of patients with two or more perceptions of excessive care and do-not-intubate and resuscitate code in the 10 participating departments over one week (pilot study conducted in July 2019)

Departments	First ward	Second ward	n(%) with 2 PECs	n(%) 2 PECs with DNIR code
1	ward 1	ward 11	3/32 (9%)	2/3 (67%)
2	ward 2	ward 9	4/19 (21%)	2/4 (50%)
3	ward 3		5/26 (19%)	1/5 (20%)
4	ward 4	ward 12	2/40 (5%)	1/2 (50%)
5	ward 5	ward 1	8/34 (24%)	2/8 (25%)
6	ward 6		2/18 (11%)	1/2 (50%)
7	ward 7	ward 1	5/58 (12%)	1/5 (20%)
8	ward 8		3/41 (7%)	2/3 (67%)
9*	ward 9	ward 13		
MICU**	ward 10		(10,2% [2])	(34% [2])
Total			32/268 (11,9%)	12/32 (37,5%)

* the incidence was not measured in one department. **The incidence in the medical ICU was retrieved from the DISPROPRICUS database.

In addition to potentially violating the basic bioethical principles, excessive care may increase the risk of physical and psychological burden in patients and relatives [5,6, 54-57]. Excessive care may also induce moral distress, compassion fatigue or avoidance behavior in clinicians [1, 5, 6, 58] with conflicts, or even worse, chronic animosity and distrust in the team [1, 5, 6, 19, 20, 58, 59] as a consequence, which will not benefit to the patient [1, 19, 20]. These issues may be even more pertinent considering the high number of patients potentially receiving excessive care against the patient or relatives' wishes, [2] or who receive care that is potentially discordant with their written-treatment-limitation decision [60]. Furthermore, postponing end-of-life decision-making and a high mortality in the ward has been associated with burnout and a higher intent to leave in clinicians [3, 5, 6, 58, 61-64]. Finally, providing excessive care has also financial consequences for the relatives [65] and the society [66-68].

In conclusion, literature and a pilot study indicate room for enhancing openness to discuss ethical sensitive issues within and between teams, and improving decision-making for the benefit of the patient at end-of-life, worldwide and more specifically in Belgium and in the Ghent University Hospital. The current intervention aims at achieving this objective by coaching doctors in self-reflective and empowering leadership, and in the management of team dynamics via repeated exploration of real patient situations triggered by clinicians on the floor.

2.3. Risk/Benefit Assessment

The intervention is directed towards doctors whereas the impact of the intervention will be measured at the patients', relatives' and clinicians' levels. Given the consequences of providing excessive care at all levels (see above) our intervention has a favorable risk to benefit ratio.

1) Only junior and senior doctors who are interested to be coached and as such consent to participate will be included in the study. Participating doctors will be coached by an experienced coach. The team meetings will be observed by an experienced clinical psychologist. Both will be supervised by a supervisor coach. The intervention is endorsed by the strategic quality cell and the medical council and is therefore completely embedded in the existing Ghent University Hospital structure. The safety, in case of for instance conflicts within the team, will be guaranty by the HR department together with the coach supervisor. Potential conflict with patients or relatives will be management as usual, by the doctor in charge or the head of the department in collaboration with the ombudsman. Finally, the majority of junior and

senior doctors and the department heads of the 10 participating departments were very enthusiastic about the overall and local DISPROPRICUS results and subsequently the rationale for this intervention during the respective meetings that were organized in Sept 2019-May 2020. They see this intervention as a real opportunity for personal development with regard to ethically distressing situation rather than as a treat.

2) One could argue that this intervention may shorten the patient’s life. Firstly, although this may be an issue, it is important to note that the primary intention of this intervention is not to shorten life but to reduce excessive care, and as such, suffering of patients with a high risk of dying. By allowing clinicians to express anonymous perceptions of excessive care, timely identification of patients potentially receiving excessive care will certainly improve (sensitivity), however the quality of decision-making will be better guaranty by enhancing reflection and decision-making in team while taking the wishes of patients and relatives into account (specificity). It is also of note that patient’s safety increases in such a setting because direct communication and negotiation between doctors and between doctors and nurses will expand. This will enhance fine-tuned decision-making for the benefit of the patient, and will reduce avoidance behavior in complex clinical situations. Secondly, previous interventions aiming at reducing excessive care in the field of palliative care, advance care planning or intensive care medicine have shown to increase the quality of life in patients at risk of dying without shortening the survival [34-38]. Because of all these reasons we decided to use written DNIR orders as primary endpoint and to consider mortality and survival with a good quality of live at home as secondary endpoints. Thirdly, treatment-limitation-decisions, at least written DNIR orders, aim at clarifying to the patients, relatives and the team what to do in case of deterioration and is therefore also not uniformly associated with mortality. Finally, we decided to power our intervention to detect an increase in written DNIR order from 35% to 50% and not higher, because this would not be a realistic expectation and because 7% of patients potentially receiving excessive care are still alive, at home with a good quality of life after 1 year [2].

2.4. Limitations

Our study has several limitations. Firstly, the participating wards were not selected at random, which may affect the external validity of our results. However, although we decided to perform this study in wards that were enthusiastic to participate, and hereby already acknowledge room for improving in ethical decision-making, we expect a high variability in ethical decision-making climates across wards as suggested by the high variability in incidence of patients potentially receiving excessive care and written DNIR order in the pilot observation (Table 1). This will enable us to assess the effect of our intervention across different ethical climates and to make recommendations with regards to our intervention for other centers. Secondly, in contrast to a drug which often has a clear biological mechanism, coaching consists of many different techniques which may have different effects in different situations. To minimize this issue our coaches will work according to the guideline in the appendix, which proposes different techniques for different situations. The different techniques and situations will also be collected during the study to perform a post-hoc exploratory analysis (see tertiary endpoints). Thirdly, the effect of the intervention may depend on the skills and experience of the coach. We decided to work with an experienced coach and an experienced clinical psychologist under supervision of a senior coach to guaranty that across all coaching situations, detailed attention is paid to all relevant aspects of the medical ethical decision-making process, and to leadership as well as productive and counterproductive group dynamics in the team. Finally, the coachability of doctors might be an issue. To encompass

that problem we will perform both an intention to coach and per protocol analysis, and will classify the doctors according to their coachability as well to be able to perform a post-hoc analysis.

3. Objectives

3.1. Primary Objectives

The primary objective of this study is to investigate whether coaching doctors in self-reflective and empowering leadership and in managing team dynamics during 4 months with regard to adult hospitalized patients potentially receiving excessive care improves ethical decision-making in comparison with standard of care. The quality of medical ethical decision-making will be assessed *objectively* via the incidence of written DNIR orders in patients potentially receiving excessive care (first primary endpoint) and *subjectively* via the EDMCQ that will be filled out by the doctors and nurses in the team (second primary endpoint). This 32-item validated questionnaire consists of 7 main domains or factors: F1 “self-reflective and empowering leadership of doctors”, F2 “open and interdisciplinary reflection”, F3 “not avoiding end-of-life decisions”, F4 “mutual respect within the interdisciplinary team”, F5 “active involvement of nurses in end-of-life care and decision-making”, F6 “active decision-making by doctors”, F7 “ethical awareness”.

These primary objectives can be formalized in the following two study hypotheses:

- The intervention changes the incidence of written DNIR order in adult hospitalized patients potentially receiving excessive care from 35% (under standard of care) to 50% over the 12-month study period.
- The intervention increases the average EDMCQ score in clinicians (doctors and nurses) by 2.8 points over the 12-month study period.

It is important to keep in mind that doctors are the only professionals who are medico-legally allowed to take written DNIR orders with or without the team (after consent of the patient or relatives) while they represent only 20% of the clinicians that will potentially fill out the EDMCQ. However, by evaluating both primary endpoints we aim to assess whether our intervention had an effect on individual decision-making by doctors or on collective decision-making in team (Fig 2). We expect that an effect on individual decision-making by doctors would affect the incidence of written DNIR orders both directly, and indirectly via F3 and F6. We expect that effect on collective decision-making in team would affect F1, which in turn affects all other EDMCQ factors and via these, the incidence of written DNIR orders.

Fig 2

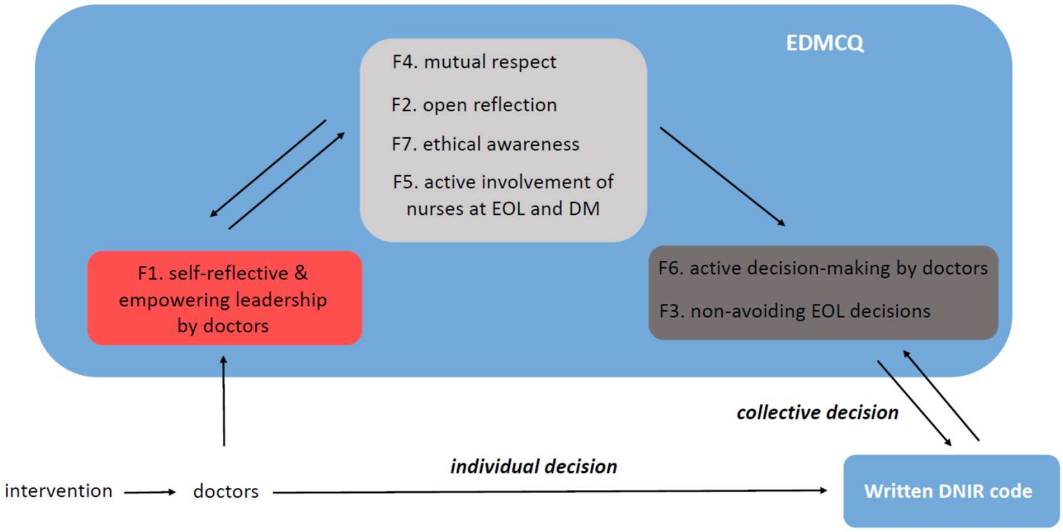

Both null hypotheses that will be tested express no change (as opposed to change).

We refer to the previous chapter (risk / benefit assessment) for the justification of the primary endpoints.

3.2. Secondary Objectives

The secondary objective of this study is to investigate whether the intervention reduces the burden of patients, relatives, clinicians and costs in comparison to standard of care.

The hypotheses are as follows:

- On patient level
    - No excess 1 year mortality (+ combined endpoint) in patients potentially receiving excessive care (effectiveness and safety)
    - Patients potentially receiving excessive care feel more involved in decision-making and rate care and communication higher (effectiveness) without impact on their psychological well-being (safety)
    - Improved quality of death and dying as reported by the nurse in patients potentially receiving excessive care who died on the ward (effectiveness)
    - Improved symptom management during hospitalization in patients potentially receiving excessive care (effectiveness)
    - Less potentially inappropriate and burdensome treatments
        - in patients potentially receiving excessive care who died on the ward (effectiveness)
        - Less ICU use in the final year of life in patients potentially receiving excessive care (effectiveness)
- On family level
    - Improved quality of death and dying reported by and less short-term psychological burden in families of patients potentially receiving excessive care who died on the ward (effectiveness and safety)

    - Families of patients potentially receiving excessive care feel more involved in decision-making and are more satisfied with care and communication (effectiveness)
- On clinician level
    - Less stress related to patients potentially receiving excessive treatment
    - Better ethical practice and less absenteeism (effectiveness)
    - Less intentional job-leave (effectiveness)
- On society level
    - Less hospital costs in patients potentially receiving excessive treatment (effectiveness)

3.3. Tertiary Objectives

To unweave the "black box effect" of coaching, the coach will collect quantitative and qualitative data of the leadership styles of the participating doctors. These styles will be used in a sub-analysis to further explore the relationship between leadership style and the primary and secondary endpoints.

In order to evaluate the quality of the implementation of coaching, the participating doctors will fill out user questionnaire and there will be focus groups to explore their experiences more in-depth.

4. End Points + Time Points

4.1. Primary End Points + Time Points

Our study uses two primary endpoints. The first is a patient-specific endpoint, defined as the occurrence of written DNIR code between hospital admission end the end of first hospital stay, which will be measured in patients potentially receiving excessive care. We focus on this endpoint because of 1) its patient centeredness 2) of its reliability as an objective endpoint that can easily be collected via the EPD and 3) our intervention is mainly focused on coaching of doctors who are medico-legally the only professionals who are allowed to formalize a DNIR with or without involvement of the team. The second is a health-care provider-specific endpoint given by the EDMCQ score, which will be assessed just before (T0) and after the 12 months study period (T3). This endpoint is team-centered and is dependent on the response rate of clinicians.

4.2. Secondary End Points + Time Points

Patients' level

We use one-year mortality as an endpoint. Because staying at home with a good quality of life is highly valued by patients, the combined one-year patient outcome in this study was defined as dead, not at home or a utility score < 0.5 [2]. For the combined endpoint, Euro-QOL-5D measures health-related quality of life, with possibility of conversion of each health state in a utility index (range -0.1584 to 1.000). This questionnaire measures health in five dimensions: mobility, self-care, usual activities, pain/discomfort, and anxiety/depression [69].

For quality of care and communication and decision-making, we use Euro-FS (European Family Satisfaction in the ICU), which is a validated 18 item questionnaire covering satisfaction with 4 domains: communication, empathy, symptom management and decision-making. [70,71].

We also use NRS score for pain as objective measure for symptom control during hospitalization. [72].

For well-being 3 weeks after discharge, we use HADS (Hospital anxiety and depression scale) which is a validated 14-item self-report assessment with subscales for anxiety and depression. Each domain has a score range of 0-21 with the following interpretation: 0-7 normal, 8-10 mild, 11-21 moderate to severe [73].

For quality of dying in patients who died on the ward, we use QODD (Quality of dying and death questionnaire). QODD-nurse: the validated Dutch version of the QODD instrument is a validated 22-item questionnaire. Euro-QODD family is a 14 item questionnaire to allow families to assess patients' quality of dying and death [74].

For the number of potentially inappropriate and burdensome treatments, we assess ventilation, dialysis, surgery, chemotherapy and radiologic diagnostics in patients who died on the ward and in patients who survived, we assess number of re-admissions, ICU use and potentially inappropriate and burdensome treatments in the final year of life in patients potentially receiving excessive care [75]

Relatives level

For quality of care and communication and decision-making, we use Euro-FS (same as patients)

For family well-being 3 weeks after discharge, we use HADS (same as in patients).

For psychological burden of families who lost their relative on the ward, we use IES-R (Impact of events scale-revised) which is a 22-item scale to self-report the frequency of intrusive and avoidant phenomena after a variety of traumatic experiences [76]. Total stress score is interpreted as follows: Low risk for PTSD (0-11), moderate risk (12-32), high risk (33 or higher).

Clinicians' level

For clinician well-being, we use stress related to perception of excessive treamtment, absenteeism (team-level) and intentional job-leave (individual level) as endpoints. These are frequently used endpoints [64].

Societal level

For hospital costs, we will assess hospital costs by the hospital billing record, and also assess hospital and ICU length of stay as well as number of diagnostic procedures (emergency department visits, hospitalizations, blood analyses, radiological investigations, surgical procedures, and chemotherapeutic and radiotherapeutics treatments)

4.3. Tertiary/Exploratory End Points

To unweave the "black box effect" of coaching, the coach will collect quantitative and qualitative data of the leadership styles of the participating doctors and the impact of these styles on the group dynamics of the team. These styles will be used in a sub-analysis to further explore the relationship between leadership style and the primary and secondary endpoints.

In order to evaluate the quality of the implementation of coaching, we added process measures based on the RE-AIM implementation framework.

-Reach: inclusion rate of doctors + number of coaching sessions

-Efficacy: evolution in scores on self-reflective and empowering leadership, comparison of competencies (self-reflective and empowering leadership and in the management of team dynamics with regard to hospitalized patients potentially receiving excessive care, see appendix) and study outcomes

-Adoption: experiences of doctors with the intervention (questionnaire + focus group)

-Implementation: experiences and satisfaction with the intervention and the coach, number of intervention elements received by and barriers and facilitators for the intervention (questionnaire + focus group)

-Maintenance: long-term adoption of the intervention (focus group and user experience questionnaire)

5. Study design

5.1. Description of study design

The study follows a stepped wedge cluster randomized trial design, run across 10 different departments of the Ghent University Hospital, representing 13 wards. All 10 departments will be randomly assigned to start a 4-month coaching period in month k=1,…,10 following a stratified design. In particular, the 3 departments with the highest incidence of written DNIR orders (based on historical data, Table 1, pg 16) will be randomly assigned to start the intervention in months 2, 4 and 6 (each time together with another ward). The 7 other wards will be randomly assigned to start the intervention according to the schematic overview in Figure 1. All wards will be followed in terms of the primary and secondary endpoints over the 10 to 12-month duration of the study.

End of Study Definition

5.1.1. For an individual subject

The subject has completed the study if he or she has completed all phases of the study, including the last phone call as described in this protocol (see section "9. Study Specific Procedures").

5.1.2. For the whole study

Overall, the end of the study is reached when the last study procedure for the last subject has occurred: last subject, last visit (LSLV).

As soon as the whole study has ended (cfr the definition above), the Coordinating Investigator shall notify the HIRUZ Clinical Trial Unit, so that the Ethics Committee can be informed in a timely manner according to the regulatory requirements (within 90 days after end of the study, or if the study had to be terminated early, this period must be reduced to 15 days and the reasons should clearly explained).

The end of study report will be submitted to the Ethics Committee, no later than 1 year after the end of the study.

5.2. Estimated duration of the study

5.2.1. For an individual subject

- Junior and senior doctors are coached for 4 months (T1-T2); timing of T1 and T2 depend on the department (cfr stepped-wedge randomization)

- Nurses, junior and senior doctors give perceptions when a patient potentially receiving excessive treatment during the whole 12 months study period (T0-T3)

- Head nurses and medical heads of department fill out questionnaire on department characteristics: 12 months (T0-T3)

- Patients and family:
    - Inclusion during their first hospitalization
    - Survey 3 weeks after discharge
    - Telephone survey 1 year after hospital discharge

5.2.2. For the whole study

FPI: T0

LSLV: one year after the end of the 10 to 12 months study period: T3 + 1 year = 22 to 24 months

6. Inclusion and Exclusion Criteria

6.1. Inclusion Criteria

- Junior and senior doctors (including medical head of department)
    - Inclusion: all junior and senior doctors taking care of hospitalized patients in the 10 participating departments of the Ghent University Hospital
- Nurses (including head nurses)
    - Inclusion: all nurses taking care of hospitalized patients in the 10 participating departments of the Ghent University Hospital
- Patients and family members
    - inclusion: first hospitalization of adult patients who are potentially receiving excessive care

6.2. Exclusion Criteria

For doctors and nurses there are no exclusion criteria.

For patients and families; persons younger than 18 years and persons who cannot understand Dutch questionnaires are excluded.

6.2.1. Screen failures

Individuals who do not meet the criteria for participation in this study (screen failure) may not be rescreened.

7. Target Population

7.1. Subjects

7.1.1. Number of subjects and planned recruitment rate

The number of subjects that will be included in this study is:

- Junior and senior doctors (including medical heads of department) : 50 à 75 junior and 50 à 75 senior doctors
- Nurses (including head nurses) : 500 à 600
- Patients and family members : 1400 to 1700

Drop-outs will not be replaced. It is expected that overall an accrual rate of 128 patients per month is realistic in the whole study.

7.1.2. Withdrawal and replacement of subjects

Subjects are free to withdraw from participation in the study at any time upon request.
In all cases, the reason why subjects are withdrawn must be recorded in detail in the eCRF and in the subject's medical records.

A subject will be considered lost to follow-up if he or she cannot be reached one year after hospital discharge by the study site staff.

The following actions must be taken if a subject fails to return to the clinic for a required study visit:

- Before a subject is deemed lost to follow-up, the investigator or designee will make every effort to regain contact with the subject (where possible, 3 telephone calls or by contacting the general practitioner). These contact attempts should be documented in the subject's medical record or study file.
- Should the subject continue to be unreachable, he or she will be considered to have withdrawn from the study with a primary reason of lost to follow-up.

7.2. Method of recruitment

- All departments within the Ghent University Hospital frequently referring patients to the ICU were invited to participate to the current study during team meetings organized in 2018-2019 aimed at discussing and reflecting about the general and local DISPRORICUS study results and the impact of providing excessive care for patients, relatives and clinicians in our hospital. All 10 departments acknowledge room to improve ethical decision-making in our hospital and were enthusiastic to participate to this study. Surgical departments did not acknowledge the need to improve ethical decision-making in our hospital. Because of financial constraints the department of pediatrics could not be included in the study but will be involved in a pilot study to assess the barriers that coaches may encounter during the intervention (Summer 2021).
- Junior and senior doctors (including medical heads of department)
    - all junior and senior doctors taking care of hospitalized patients in the 10 participating departments of the Ghent University Hospital will be informed about the study on their medical staff meeting, we will give our contact details in order to ask individual questions after the meeting
    - those who give informed consent will be included in the study
    - by means of coaching, participants get the opportunity for personal growth, which is the only (but important) incentive
- Nurses (including head nurses)
    - Inclusion: all nurses taking care of hospitalized patients in the 10 participating departments of the Ghent University Hospital will be informed about the study on their nursing staff meeting, we will give our contact details in order to ask individual questions after the meeting
    - those who give informed consent will be included in the study
    - there is no financial incentive, from former studies (APPROPRICUS and DISPROPRICUS) we learnt that nurses were very eager to give their anonymous perception about the ethical climate and their perception of excessive care

- Patients and family members
    - inclusion: adult patients (or their legal representative in case of incompetence) identified as potentially receiving excessive care will be asked informed consent by the treating physician to fill-out survey 3 weeks after discharge and to be contacted 1 year after discharge. They will be asked to indicate 1 family member to be contacted.
    - That family member will be asked informed consent also during hospitalization
    - There is no financial incentives for study participation
    - The investigator or designee will make every effort to regain contact with the subject after 1 year to collect living situation 1 year after discharge (where possible, 3 telephone calls or by contacting the general practitioner).

Since our intervention is completely embedded in the hospital, we will be able to count on the communication department of the Ghent University Hospital to empower clinicians to participate by means of posters, flyers and multimedia.

7.3. Screening

Every patient that is identified by clinicians as potentially receiving excessive care.

8. Study Specific Procedures

8.1. Randomisation/blinding

- Junior and senior doctors (including medical heads of department)
  - all junior and senior doctors taking care of hospitalized patients in the 10 participating departments of the Ghent University Hospital will be informed about the study on their medical staff meeting, we will give our contact details in order to ask individual questions after the meeting
  - they are free in how they accept the offered coaching
  - they will each be given an electronic link to fill-out the RED-CAP EDMCQ at T1 online and are free to participate or not
  - filling-out the perceptions when a patient potentially receiving excessive treatment will be seen as consent to participate

- Nurses (including head nurses)
  - all nurses taking care of hospitalized patients in the 10 participating departments of the Ghent University Hospital will be informed about the study on their nursing staff meeting, we will give our contact details in order to ask individual questions after the meeting
  - they will each be given an electronic link to fill-out the RED-CAP EDMCQ at T1 online and are free to participate or not
  - filling-out the when a patient potentially receiving excessive treatment perceptions will be seen as consent to participate

- Patients and family members
  - All adult patients (or their legal representative in case of incompetence) identified as potentially receiving excessive care will be asked informed consent by the treating physician to fill-out survey 3 weeks after discharge and to be contacted 1 year after discharge. They will be asked to indicate 1 family member to be contacted.
  - That family member will be asked informed consent also during hospitalization
  - There are no financial incentives for study participation
  - The investigator or designee will make every effort to regain contact with the subject after 1 year to collect living situation 1 year after discharge (where possible, 3 telephone calls or by contacting the general practitioner).

8.2. Randomisation/blinding

Randomisation of the 10 departments will be performed by the Ghent University Department of Applied Mathematics, Computer Science and Statistics based on a random number generator in the software R. As in nearly all stepped wedge designs, the nature of the intervention is such that it cannot be blinded to health care providers. However, patients will be blind to the intervention.

8.3. Overview of collected data

OUTCOME DOMAIN	OUTCOMES	INSTRUMENT	DATA SOURCE	TIMING OF MEASUREMENT
**ETHICAL DECISION-MAKING (PRIMARY ENDPOINTS)**				
**ADVANCE CARE PLANNING**	DNIR incidence		Chart extraction	At the end of first hospital stay
**TEAM PERCEPTION OF ETHICAL CLIMATE**	Ethical decision-making climate	EDMCQ	Nurses and physicians	T0 and T3
**PATIENT-CENTERED OUTCOME**				
**IN ALL PATIENTS**				
**LIVING SITUATION AT 1 YEAR**	(Time from 2PEC to) survival, QOL and place of residence in survivors	Euro-QOL+survey	Patient/family by telephone call	1 year after first hospital stay
**QUALITY OF CARE, COMMUNICATION AND DECISION-MAKING**	Pain	NRS	Chart extraction	At the end of the first hospital stay
	Symptom management	Euro-FS adapted for the patient (part 1)	Patient discharged alive	3 weeks after discharge
	Symptom management	Euro-FS (part 1)	Family	3 weeks after discharge
	Quality of communication and decision-making	Euro-FS adapted for the patient (part 2)	Patient discharged alive	3 weeks after discharge
**WELL-BEING AFTER DISCHARGE**	Anxiety and depression	HADS	Patient discharged alive	3 weeks after discharge
**IN PATIENTS WHO DIED ON THE WARD**				
**QUALITY OF DYING**	Quality of dying	QODD-nurse	Nurse	1 week afterdischarge
	Quality of dying	Euro-QODD	Family	3 weeks after discharge
	Potentially inappropriate treatments at EOL		Chart extraction: ICU stay, surgery, chemotherapy, radiotherapy	At the end of first hospital stay

OUTCOME DOMAIN	OUTCOMES	INSTRUMENT	DATA SOURCE	TIMING OF MEASUREMENT
**FAMILY OUTCOME**				
**IN PATIENTS WHO WERE DISCHARGED ALIVE**				
**WELL-BEING AFTER DISCHARGE**	Anxiety and depression	HADS	Family	3 weeks after discharge
**QUALITY OF COMMUNICATION AND DECISION-MAKING**	Quality of communication and decision-making	Euro-FS (part 2)	Family	3 weeks after discharge
**IN PATIENTS WHO DIED ON THE WARD**				
**WELL-BEING AFTER DISCHARGE**	Anxiety and depression	HADS	Family	3 weeks after discharge
	Post-traumatic stress	IES-R	Family	3 weeks after discharge
**QUALITY OF COMMUNICATION AND DECISION-MAKING**	Quality of communication and decision-making	Euro-FS (part 2)	Family	3 weeks after discharge
**HEALTHCARE COSTS**				
	Cost of the intervention			
	Payer’s hospitalization cost		Hospital billing record	At the end of first hospital admission
	Hospital (and ICU) length of stay and diagnostics		Chart extraction	At the end of first hospital admission
	Hospital re-admission, ICU and burdensome treatments rates after discharge		Patient/family by telephone call	1 year after the first hospitalisation
**WELL-BEING OF HEALTH CARE PROFESSIONALS**				
	Stress related to perception of excessive treatment		Nurses and physicians	T0 to T3
	Intention to leave job		Nurses and physicians	T0 and T3
**TEAM WELL-BEING AND PERFORMANCE**				
	Absenteeism		HR department	T0 and T3
	Ethical practice score	Ethical practice score	Head nurse	T0 and T3

OUTCOME DOMAIN	OUTCOMES	INSTRUMENT	DATA SOURCE	TIMING OF MEASUREMENT
**PROCESS MEASURES**				
**NUMBER OF COACHING SESSIONS)**			Survey (by coach)	During intervention
**SELF-REFLECTIVE AND EMPOWERING LEADERSHIP SKILLS**		Survey on quality of self-reflection	Coach	During intervention
		Survey on quality of empowering leadership	Observator of interdisiplinary team meeting	During intervention
			Survey (user experience by individual doctors)	T2
			Focus groups in doctors	T2

8.4. Schematic overview of the data collection & interventions

**Procedures**	**Inclusion during hospitalization**	**3 weeks post-discharge**	**Follow Up 1 year post-discharge**
Informed consent	X		
Survey		X	X

9. Statistical Considerations

9.1. Sample size calculation

The outcome(s) on which the sample size calculation is based upon are the incidence of written DNIR order and the EDMCQ.

For written DNIR code, analysis will be based on logistic mixed effect models with random intercept to account for between-department variability, assuming a constant risk before and after intervention, and a linearly changing risk during the intervention. Based on such analysis, a Monte Carlo power evaluation showed that under the considered stratified randomised design, a Wald test at the 5% significance level delivers 86% power to detect an intervention effect when data are available for 5 patients potentially receiving excessive treatment per department per month (over a period of 12 months), if the risk of written DNIR order in adults patients potentially receiving excessive treatment increases from 35% before to 50% after intervention; with an incidence of patients potentially receiving treatment equaling 12% (Table 1, pg 16), this amounts to approximately 42 patients per department per month. Independently from each other, respectively the scientific experts in the field of coaching and the experienced coach of our steering committee estimated that a 4 month intervention would be required to detect an effect in the team and at least 8 coaching sessions to detect an effect at the individual level.

The above calculation amounts to a total of 5000 patients, of which 600 (i.e., 12%) patients potentially receiving excessive treatment over a 12 month period. Based on our measurement (268 patients over 9 departments in one week of whom 32 who potentially received excessive treatment; thus 1980 patients potentially receiving excessive treatment over the 10 participating department, which we reduced to a total of 1700 to adjust for patients potentially receiving excessive treatment with a length of stay of more than 1 week in July 2019, Figure 1), this suggests sufficient power to detect a difference in the primary endpoint and probably also in many secondary endpoints. In the above power analysis, we considered a design in which all participating departments are randomly assigned to start a 4-month coaching intervention in either month 1, 2, 3, ... or 7 following the previously described stratified design. Moreover, we assumed an intra-class correlation of 0.025. This means that for 95% of hospital departments, the risk of written DNIR order lies between 25% and 47% before intervention, and between 38% and 62% after intervention.

In a secondary analysis, we will additionally adjust for a fixed linear time slope to account for period effects, even though no such effects are expected. With 5 patients with potentially receiving excessive treatment per department per month (over a period of 12 months), this secondary analysis has 35% power at the 5% significance level to detect an intervention effect if the risk of written DNIR order in patients potentially receiving excessive treatment increases from 35% before to 50% after intervention. In the more likely event that we observe 13 patients potentially receiving excessive treatment per department per month (over a period of 12 months), which is what we expect, the power for this secondary analysis would increase to 75%.

For the EDMCQ, the sample size calculation was based on linear mixed models for the change in EDMCQ score after versus before the intervention, including a random intercept to account for between-department variability and a random intercept to account for variability between health care providers. Based on such analysis, a Monte Carlo power evaluation showed that a Wald test at the 5% significance level delivers 93% power to detect an intervention effect when data are available for 5 health care providers per unit, if the EDMCQ score increases on average with 2.8 units. For this, we assumed an intra-unti correlation of 0.14, an intra-HCP

correlation of 0.25 and a total standard deviation of 5.03. Additionally adjusting for a fixed linear time slope to account for period effects reduces power to 39%.

9.2. Type of statistical methods

Primary analyses will follow the intention-to-treat principle. Analysis of the incidence of written DNIR order will be based on logistic mixed effects models with random intercept to account for between-department, assuming a constant risk before and after intervention, and a linearly changing risk during the intervention.

While period effects are not expected, we will examine evidence for period effects in a secondary analysis by including a fixed linear time slope in the model; we will report the corresponding adjusted intervention effect. In a subsequent secondary analysis, we will use instrumental variable methods to account for noncompliance either due to clinicians not attending all coaching sessions, or due to clinicians previously having attended coaching sessions when they worked in a different department. In such analyses, we will in a first stage fit a linear model to predict the percentage of planned coaching sessions attended by clinicians in function of time and randomized arm (which may exceed 1 for junior doctors who attended sessions previously in a different department). In a second stage, a logistic mixed effects model will be fitted for the incidence of written DNIR order in function of time and the first-stage prediction. The coefficient of this first-stage prediction then expresses the effect of attending all coaching sessions under perfect adherence.

Analysis of the change in ECDMQ score will be based on linear mixed effects models for the ECDMQ score, an intervention indicator (1 if the clinician was already exposed to the intervention and 0 otherwise), a random intercept to account for between-department variability and a nested random intercept to account for between-clinician variability.

For secondary endpoints, the analysis of continuous endpoints will be based on linear mixed models and the analysis of dichotomous endpoints on logistic mixed models, each time including a random intercept at the ward level to account for between-department variability. Analyses at the patient level will include a fixed effect of time to correct for period effects, if there is evidence for such effects at the 5% significance level. Analyses at the clinician level will instead include a nested random intercept to account for between-clinician variability.

9.3. Statistical analysis team

Analysis of the primary and secondary endpoints will be done by the Department of Applied Mathematics, Computer Science and Statistics of Ghent University (responsible: Prof. Stijn Vansteelandt).

9.4. Interim analysis

No interim analysis is planned.

10. Data handling

10.1. Method of data collection

Subjects that are included in the study, will be assigned a unique study number upon their registration in REDCap. The subject identification list will be safeguarded by the site. The name and any other directly identifying details will not be included in the study database.

10.1.1. Case Report Form

An electronic data capture (EDC) system, i.e. REDCap, will be used for data collection. Data reported on each eCRF should be consistent with the source data. If information is not known, this must be clearly indicated on the eCRF. All missing and ambiguous data will be clarified.

Only the data required by the protocol are captured in the eCRF. The eCRFs and the database will be developed, based on the protocol. The final eCRF design will be approved by the Coordinating Investigator.

All data entries and corrections will only be performed by study site staff, authorized by the investigator. Data will be checked by trained personnel (data manager) and any errors or inconsistencies will be clarified. The investigator must verify that all data entries in the eCRF are accurate and correct.

REDCap is provided and maintained by Vanderbilt University; a license for use was granted to the Health, Innovation and Research Institute (HIRUZ). REDCap is a web-based system. The study site staff is responsible for data entry in REDCap.

10.2. Data storage

The data is accessed through a web browser directly on the secure REDCap server. The server is hosted within the UZ Gent campus and meets hospital level security and back-up requirements.

Privacy and data integrity between the user's browser and the server is provided by mandatory use of Transport Layer Security (TLS), and a server certificate issued by TERENA (Trans-European Research and Education Networking Association). All study sites will have access to REDCap. Site access is controlled with IP restriction.

10.3. Archiving of data

The investigator and sponsor specific essential documents will be retained for at least 20 years. At that moment, it will be judged whether it is necessary to retain them for a longer period, according to applicable regulatory or other requirement(s).

10.4. Access to data

Direct access will be granted to authorised representatives from the Sponsor, host institution and the regulatory authorities to permit study-related monitoring, audits and inspections.

Login in REDCap is password controlled. Each user will receive a personal login name and password and will have a specific role which has predefined restrictions on what is allowed in REDCap. Furthermore, users will only be able to see data of subjects of their own site. Any activity in the software is traced and transparent via the audit trail and log files.

11. Monitoring/Auditing/Inspection

11.1. Monitoring

Not applicable

11.2. Inspection

This study can be inspected at any time by regulatory agencies during or after completion of the study. Therefore access to all study records, including source documents, must be accessible to the inspection representatives. Subject privacy must be respected at all times, in accordance to GDPR, GCP and all other applicable local regulations.

11.3. Protocol Deviation policy

Sponsor and all investigators agree to take any reasonable actions to correct protocol deviations/violations noted during inspection. All deviations must be documented on a protocol deviation log by the study team that is kept available at any time for inspection purposes. Under emergency circumstances, deviations from the protocol to protect the rights, safety or well-being of human subjects may proceed without prior approval of the sponsor and the EC.

11.4. Serious breach to GCP and/or the protocol

Critical issues that significantly affect patient safety, data integrity and/or study conduct should be clearly documented and will be communicated with HIRUZ CTU and possibly both the applicable Ethics Committee.

Please contact HIRUZ CTU asap in case of a serious breach: hiruz.ctu@uzgent.be and/or +3293320500.

Early determination of the study may be necessary in case of major non-compliance.

12. Ethical and legal aspects

12.1. Good Clinical Practice

The study will be conducted conform the latest version of the ICH E6 (R2) GCP guidelines, creating a standard for the design, conduct, performance, monitoring, auditing, recording, analyses and reporting of clinical studies that provides assurance that the data and reported results are accurate and that the rights, integrity and confidentiality of study subjects are protected.

12.2. Informed Consent

Eligible subjects may only be included in the study after providing written (witnessed, if needed) Ethics Committee-approved informed consent, or, if incapable of doing so, after such consent has been provided by a legally acceptable representative(s) of the subject.
Informed consent must be obtained before conducting any study-specific procedures (as described in this protocol).

Prior to entry in the study, the investigator must explain to potential subjects or their legal representatives the study and the implication of participation. Subjects will be informed that their participation is voluntary and that they may withdraw consent to participate at any time. Participating subjects will be told that their records may be accessed by competent authorities and by authorized persons without violating the confidentiality of the subject, to the extent permitted by the applicable law(s) and/or regulations. By signing the Informed Consent Form (ICF), the subjects or legally acceptable representatives are authorizing such access.

After this explanation and before entry to the study, written, dated and signed informed consent should be obtained from the subject or legally acceptable representative. The ICF should be provided in a language sufficiently understood by the subject. Subjects must be given the opportunity to ask questions.

The subject or legally acceptable representative will be given sufficient time to read the ICF and to ask additional questions. After this explanation and before entry to the study, consent should be appropriately recorded by means of either the subject's or his/her legal representative's dated signature or the signature of an independent witness who certifies the subject's consent in writing. After having obtained the consent, a copy of the ICF must be given to the subject.

In case the subject or legally acceptable representative is unable to read, an impartial witness must attest the informed consent.

Subjects who are unable to comprehend the information provided can only be enrolled after consent of a legally acceptable representative.

The following information should be added to the electronic patient dossier (EPD):

- which version of the ICF was obtained
- who signed the ICF
- if sufficient time has been given to consider participation into the study
- which investigator obtained ICF with the date of signature
- if a copy was provided to the patient
- start and end of participation in the study

12.3. Approval of the study protocol

12.3.1. General

The protocol has been reviewed and approved by the Ethics Committee of the Ghent University (Hospital).

12.3.2. Protocol amendments

Any significant change or addition to the protocol can only be made in a written protocol amendment that must be approved by the Ethics Committee.

Only amendments that are intended to eliminate an apparent immediate safety threat to patients may be implemented immediately.

Notwithstanding the need for approval of formal protocol amendments, the investigators are expected to take any immediate action, required for the safety of any subject included in this study, even if this action represents a deviation from the protocol. These actions should always be notified to the sponsor.

12.4. Confidentiality and Data Protection

All study data will be handled in accordance with the law on General Data Protection Regulation (GDPR) and institutional rules (in Belgium: in accordance with the Belgian laws dated on 30 July 2018 and 22 August 2002).

The collection and processing of personal data from subjects enrolled in this study will be limited to those data that are necessary to fulfill the objectives of the study. These data must be collected and processed with adequate precautions to ensure confidentiality and compliance with applicable data privacy protection laws and regulations.
Appropriate technical and organizational measures to protect the personal data against unauthorized disclosures or access, accidental or unlawful destruction, or accidental loss or alteration must be put in place. Sponsor and site personnel whose responsibilities require access to personal data agree to keep the identity of subjects confidential.

The informed consent obtained from the subject includes explicit consent for the processing of personal data and for the investigator/institution to allow direct access to his or her original medical records (source data/documents) for study-related Ethics Committee review and regulatory inspection. This consent also addresses the transfer of the data to other entities, if applicable.

12.5. Liability and Insurance

The sponsor has taken a no fault insurance for this study (applicable in Belgium), in accordance with the relevant legislation (article 29, Belgian Law of May 7, 2004).

Sponsor: Ghent University Hospital

Insurance Details: Allianz Global Corporate & Specialty, Uitbreidingstraat 86, 2600 Berchem, Belgium, tel: +32 33 04 16 00

Policy number: BEL001889

12.6. End of Study Notification

If all subjects have completed the study, a notification of the end of the study should be submitted to the Ethics Committee. This notification should be made within 90 days of the end of the clinical study. In case of early termination (definition in CT-1, 4.2), this is reduced to 15 days.

13. Publication policy

This study will be registered at ClinicalTrials.gov, and results information from this study will be submitted to ClinicalTrials.gov. In addition, every attempt will be made to publish results in peer-reviewed journals.

The local investigators are responsible for recruiting patients according to the inclusion and exclusion criteria, collecting informed consent and empowering teams to provide PECs during the entire 12 months study period together with the study nurse, coach, communication department of the Ghent University Hospital and the principal investigators.

All local investigators who fulfill the authorship requirements will be coauthor of at least one of the principal publications. In case the journal does not allow a high number of coauthors (members of the steering committee + local investigators of 10 wards), co-authorship will be divided among the first main publications. Local investigators may subsequently become first authors of publications on CODE study sub-analyses once the initial main results have been published.

Professional medical writers will not be hired. Every person who collaborated with this study outside the steering committee and the list of local investigators will be acknowledged in the publications. Funding will be acknowledged appropriately.

14. Reference List

1) Ethical decision-making climate in the ICU: theoretical framework and validation of a self-assessment tool. Van den Bulcke B, Piers R, Jensen HI, Malmgren J, Metaxa V, Reyners AK, Darmon M, Rusinova K, Talmor D, Meert AP, Cancelliere L, Zubek L, Maia P, Michalsen A, Decruyenaere J, Kompanje EJO, Azoulay E, Meganck R, Van de Sompel A, Vansteelandt S, Vlerick P, Vanheule S, Benoit DD.Van den Bulcke B, et al. BMJ Qual Saf. 2018 Oct;27(10):781-789. doi: 10.1136/bmjqs-2017-007390.

2) Outcome in patients perceived as receiving excessive care across different ethical climates: a prospective study in 68 intensive care units in Europe and the USA. Benoit DD, Jensen HI, Malmgren J, Metaxa V, Reyners AK, Darmon M, Rusinova K, Talmor D, Meert AP, Cancelliere L, Zubek L, Maia P, Michalsen A, Vanheule S, Kompanje EJO, Decruyenaere J, Vandenberghe S, Vansteelandt S, Gadeyne B, Van den Bulcke B, Azoulay E, Piers RD; DISPROPRICUS study group of the Ethics Section of the European Society of Intensive Care Medicine. Intensive Care Med. 2018 Jul;44(7):1039-1049. doi: 10.1007/s00134-018-5231-8.

3) Perceptions of appropriateness of care among European and Israeli intensive care unit nurses and physicians. Piers RD, Azoulay E, Ricou B, Dekeyser Ganz F, Decruyenaere J, Max A, Michalsen A, Maia PA, Owczuk R, Rubulotta F, Depuydt P, Meert AP, Reyners AK, Aquilina A, Bekaert M, Van den Noortgate NJ, Schrauwen WJ, Benoit DD; APPROPRICUS Study Group of the Ethics Section of the ESICM. JAMA. 2011 Dec 28;306(24):2694-703. doi: 10.1001/jama.2011.1888.

4) Mortality prediction models, causal effects, and end-of-life decision making in the intensive care unit. Maley JH, Wanis KN, Young JG, Celi LA. BMJ Health Care Inform. 2020 Oct;27(3):e100220. doi: 10.1136/bmjhci-2020-100220.

5) Causes and consequences of disproportionate care in intensive care medicine. Kompanje EJ, Piers RD, Benoit DD. Curr Opin Crit Care. 2013 Dec;19(6):630-5. doi: 10.1097/MCC.0000000000000026.

6) Ethics and end-of-life care for adults in the intensive care unit. Curtis JR, Vincent JL.Curtis JR, et al. Lancet. 2010 Oct 16;376(9749):1347-53. doi: 10.1016/S0140-6736(10)60143-2. Epub 2010 Oct 11.

7) An Official ATS/AACN/ACCP/ESICM/SCCM Policy Statement: Responding to Requests for Potentially Inappropriate Treatments in Intensive Care Units. Bosslet GT, Pope TM, Rubenfeld GD, Lo B, Truog RD, Rushton CH, Curtis JR, Ford DW, Osborne M, Misak C, Au DH, Azoulay E, Brody B, Fahy BG, Hall JB, Kesecioglu J, Kon AA, Lindell KO, White DB; American Thoracic Society ad hoc Committee on Futile and Potentially Inappropriate Treatment; American Thoracic Society; American Association for Critical Care Nurses; American College of Chest Physicians; European Society for Intensive Care Medicine; Society of Critical Care. Am J Respir Crit Care Med. 2015 Jun 1;191(11):1318-30. doi: 10.1164/rccm.201505-0924ST.

8) Uncertainty--the other side of prognosis. Smith AK, White DB, Arnold RM. N Engl J Med. 2013 Jun 27;368(26):2448-50.

9) Changes in End-of-Life Practices in European Intensive Care Units From 1999 to 2016. Sprung CL, Ricou B, Hartog CS, Maia P, Mentzelopoulos SD, Weiss M, Levin PD, Galarza L, de la Guardia V, Schefold JC, Baras M, Joynt GM, Bülow HH, Nakos G, Cerny V, Marsch S, Girbes AR, Ingels C, Miskolci O, Ledoux D, Mullick S, Bocci MG, Gjedsted J, Estébanez B, Nates JL, Lesieur O, Sreedharan R, Giannini AM, Fuciños LC, Danbury CM, Michalsen A, Soliman IW, Estella A, Avidan A. JAMA. 2019 Oct 2;322(17):1-12. doi: 10.1001/jama.2019.

10) Assessment of Variability in End-of-Life Care Delivery in Intensive Care Units in the United States. Kruser JM, Aaby DA, Stevenson DG, Pun BT, Balas MC, Barnes-Daly MA, Harmon L, Ely EW. JAMA Netw Open. 2019 Dec 2;2(12):e1917344. doi: 10.1001/jamanetworkopen.2019.17344.

11) Global variability in withholding and withdrawal of life-sustaining treatment in the intensive care unit: a systematic review. Mark NM, Rayner SG, Lee NJ, Curtis JR. Intensive Care Med. 2015 Sep;41(9):1572-85. doi: 10.1007/s00134-015-3810-5. Epub 2015 Apr 23.

12) Comparison of Site of Death, Health Care Utilization, and Hospital Expenditures for Patients Dying With Cancer in 7 Developed Countries. Bekelman JE, Halpern SD, Blankart CR, Bynum JP, Cohen J, Fowler R, Kaasa S, Kwietniewski L, Melberg HO, Onwuteaka-Philipsen B, Oosterveld-Vlug M, Pring A, Schreyögg J, Ulrich CM, Verne J, Wunsch H, Emanuel EJ; International Consortium for End-of-Life Research (ICELR). JAMA. 2016 Jan 19;315(3):272-83. doi: 10.1001/jama.2015.18603.

13) Trends in the aggressiveness of end-of-life cancer care in the universal health care system of Ontario, Canada. Ho TH, Barbera L, Saskin R, Lu H, Neville BA, Earle CC. J Clin Oncol. 2011 Apr 20;29(12):1587-91. doi: 10.1200/JCO.2010.31.9897. Epub 2011 Mar 14.

14) Place of death in the population dying from diseases indicative of palliative care need: a cross-national population-level study in 14 countries. Pivodic L, Pardon K, Morin L, Addington-Hall J, Miccinesi G, Cardenas-Turanzas M, Onwuteaka-Philipsen B, Naylor W, Ruiz Ramos M, Van den Block L, Wilson DM, Loucka M, Csikos A, Rhee YJ, Teno J, Deliens L, Houttekier D, Cohen J; EURO IMPACT. J Epidemiol Community Health. 2016 Jan;70(1):17-24. doi: 10.1136/jech-2014-205365. Epub 2015 Jul 22.J Epidemiol Community Health. 2016. PMID: 26202254

15) International study of the place of death of people with cancer: a population-level comparison of 14 countries across 4 continents using death certificate data. Cohen J, Pivodic L, Miccinesi G, Onwuteaka-Philipsen BD, Naylor WA, Wilson DM, Loucka M, Csikos A, Pardon K, Van den Block L, Ruiz-Ramos M, Cardenas-Turanzas M, Rhee Y, Aubry R, Hunt K, Teno J, Houttekier D, Deliens L. Br J Cancer. 2015 Nov 3;113(9):1397-404. doi: 10.1038/bjc.2015.312. Epub 2015 Sep 1.

16) Place of Death among Hospitalized Patients with Cancer at the End of Life. Wallace SK, Waller DK, Tilley BC, Piller LB, Price KJ, Rathi N, Haque S, Nates JL. J Palliat Med. 2015 Aug;18(8):667-76. doi: 10.1089/jpm.2014.0389. Epub 2015 Apr 30.

17) Provider and Patient Gender Influence on Timing of Do-Not-Resuscitate Orders in Hospitalized Patients with Cancer. Crosby MA, Cheng L, DeJesus AY, Travis EL, Rodriguez MA. J Palliat Med. 2016 Jul;19(7):728-33. doi: 10.1089/jpm.2015.0388. Epub 2016 May 9.

18) Coping With Moral Distress in Oncology Practice: Nurse and Physician Strategies. Lievrouw A, Vanheule S, Deveugele M, Vos M, Pattyn P, Belle V, Benoit DD. Oncol Nurs Forum. 2016 Jul 1;43(4):505-12. doi: 10.1188/16.ONF.505-512

19) The inner life of physicians and care of the seriously ill. Meier DE, Back AL, Morrison RS. JAMA. 2001 Dec 19;286(23):3007-14. doi: 10.1001/jama.286.23.3007.

20) The Inner Lives of Doctors: Physician Emotion in the Care of the Seriously Ill. Childers J, Arnold B. Am J Bioeth. 2019 Dec;19(12):29-34.

21) Containing Anxiety in Institutions: Selected Essays. Free Association Books London, 1988 Isabel E. P. Menzies- Lyth

22) Clinicians' Perceptions of Futile or Potentially Inappropriate Care and Associations with Avoidant Behaviors and Burnout. Chamberlin P, Lambden J, Kozlov E, Maciejewski R, Lief L, Berlin DA, Pelissier L, Yushuvayev E, Pan CX, Prigerson HG. J Palliat Med. 2019 Sep;22(9):1039-1045.

23) Theory and practice of clinical ethics support services: narrative and hermeneutical perspectives. Porz R, Landeweer E, Widdershoven G. Bioethics. 2011 Sep;25(7):354-60. doi: 10.1111/j.1467-8519.2011.01911.x.

24) Ethical climate in contemporary paediatric intensive care. Moynihan KM, Taylor L, Crowe L, Balnaves MC, Irving H, Ozonoff A, Truog RD, Jansen M. J Med Ethics. 2021 Jan 11:medethics-2020-106818. doi: 10.1136/medethics-2020-106818.

25) Developing Physician Leaders: A Perspective on Rationale, Current Experience, and Needs. Stoller JK. Chest. 2018 Jul;154(1):16-20. doi: 10.1016/j.chest.2017.12.014.

26) Inappropriate care in European ICUs: confronting views from nurses and junior and senior physicians. Piers RD, Azoulay E, Ricou B, DeKeyser Ganz F, Max A, Michalsen A, Azevedo Maia P, Owczuk R, Rubulotta F, Meert AP, Reyners AK, Decruyenaere J, Benoit DD; Appropricus Study Group of the Ethics Section of the European Society of Intensive Care Medicine. Chest. 2014 Aug;146(2):267-275. doi: 10.1378/chest.14-0256.

27) Perceptions of Ethical Decision-Making Climate Among Clinicians Working in European and U.S. ICUs: Differences Between Nurses and Physicians. Jensen HI, Hebsgaard S, Hansen TCB, Johnsen RFA, Hartog CS, Soultati I, Szucs O, Wilson ME, van den Bulcke B, Benoit DD, Piers R. Crit Care Med. 2019 Dec;47(12):1716-1723.

28) Physician-related barriers to communication and patient- and family-centred decision-making towards the end of life in intensive care: a systematic review. Visser M, Deliens L, Houttekier D. Crit Care. 2014 Nov 18;18(6):604. doi: 10.1186/s13054-014-0604-z

29) "Futile" care: do we provide it? Why? A semistructured, Canada-wide survey of intensive care unit doctors and nurses. Palda VA, Bowman KW, McLean RF, Chapman MG. J Crit Care. 2005 Sep;20(3):207-13. doi: 10.1016/j.jcrc.2005.05.006.

30) Interprofessional Shared Decision-Making in the ICU: A Systematic Review and Recommendations From an Expert Panel. Michalsen A, Long AC, DeKeyser Ganz F, White DB, Jensen HI, Metaxa V, Hartog CS, Latour JM, Truog RD, Kesecioglu J, Mahn AR, Curtis JR. Crit Care Med. 2019 Sep;47(9):1258-1266.

31) Leadership Essentials for the Chest Physician: Models, Attributes, and Styles. Stoller JK. Chest. 2020 Sep 19:S0012-3692(20)34516-5. doi: 10.1016/j.chest.2020.09.095.

32) Leadership Essentials for the Chest Physician: Emotional Intelligence. Stoller JK. Chest. 2020 Sep 19:S0012-3692(20)34514-1. doi: 10.1016/j.chest.2020.09.093.

33) "How I Do It": Building Teams in Health Care. Stoller JK. Chest. 2020 Sep 21:S0012-3692(20)34513-X. doi: 10.1016/j.chest.2020.09.092.

34) Randomized Trial of Communication Facilitators to Reduce Family Distress and Intensity of End-of-Life Care. Curtis JR, Treece PD, Nielsen EL, Gold J, Ciechanowski PS, Shannon SE, Khandelwal N, Young JP, Engelberg. Am J Respir Crit Care Med. 2016 Jan 15;193(2):154-62. doi: 10.1164/rccm.201505-0900OC

35) A Randomized Trial of a Family-Support Intervention in Intensive Care Units. White DB, Angus DC, Shields AM, Buddadhumaruk P, Pidro C, Paner C, Chaitin E, Chang CH, Pike F,

Weissfeld L, Kahn JM, Darby JM, Kowinsky A, Martin S, Arnold RM; PARTNER Investigators. N Engl J Med. 2018 Jun 21;378(25):2365-2375. doi: 10.1056/NEJMoa1802637. Epub 2018 May 23.

36) Association Between Palliative Care and Patient and Caregiver Outcomes: A Systematic Review and Meta-analysis. Kavalieratos D, Corbelli J, Zhang D, Dionne-Odom JN, Ernecoff NC, Hanmer J, Hoydich ZP, Ikejiani DZ, Klein-Fedyshin M, Zimmermann C, Morton SC, Arnold RM, Heller L, Schenker Y. JAMA. 2016 Nov 22;316(20):2104-2114. doi: 10.1001/jama.2016.16840.

37) Effect of early and systematic integration of palliative care in patients with advanced cancer: a randomised controlled trial. Vanbutsele G, Pardon K, Van Belle S, Surmont V, De Laat M, Colman R, Eecloo K, Cocquyt V, Geboes K, Deliens L. Lancet Oncol. 2018 Mar;19(3):394-404. doi: 10.1016/S1470-2045(18)30060-3.

38) Early palliative care for patients with metastatic non-small-cell lung cancer. Temel JS, Greer JA, Muzikansky A, Gallagher ER, Admane S, Jackson VA, Dahlin CM, Blinderman CD, Jacobsen J, Pirl WF, Billings JA, Lynch TJ. N Engl J Med. 2010 Aug 19;363(8):733-42. doi: 10.1056/NEJMoa1000678

39) Effect of ethics consultations on nonbeneficial life-sustaining treatments in the intensive care setting: a randomized controlled trial. Schneiderman LJ, Gilmer T, Teetzel HD, Dugan DO, Blustein J, Cranford R, Briggs KB, Komatsu GI, Goodman-Crews P, Cohn F, Young EW. JAMA. 2003 Sep 3;290(9):1166-72. doi: 10.1001/jama.290.9.1166

40) Leadership development programs for physicians: a systematic review. Frich JC, Brewster AL, Cherlin EJ, Bradley EH. J Gen Intern Med. 2015 May;30(5):656-74. doi: 10.1007/s11606-014-3141-1.

41) Hospitalizations of cancer patients in the last month of life: quality indicator scores reveal large variation between four European countries in a mortality follow-back study. De Roo ML, Francke AL, Van den Block L, Donker GA, Alonso JE, Miccinesi G, Moreels S, Onwuteaka-Philipsen BD, Salvetti A, Deliens L; EURO IMPACT. BMC Palliat Care. 2014 Nov 27;13:54. doi: 10.1186/1472-684X-13-54. eCollection 2014

42) Site of Death, Place of Care, and Health Care Transitions Among US Medicare Beneficiaries, 2000-2015. Teno JM, Gozalo P, Trivedi AN, Bunker J, Lima J, Ogarek J, Mor V. JAMA. 2018 Jul 17;320(3):264-271. doi: 10.1001/jama.2018.8981

43) Trends in Noninvasive and Invasive Mechanical Ventilation Among Medicare Beneficiaries at the End of Life. Sullivan DR, Kim H, Gozalo PL, Bunker J, Teno JM. JAMA Intern Med. 2021 Jan 1;181(1):93-102. doi: 10.1001/jamainternmed.2020.5640.

44) Heterogeneity and changes in preferences for dying at home: a systematic review. Gomes B, Calanzani N, Gysels M, Hall S, Higginson IJ. BMC Palliat Care. 2013 Feb 15;12:7. doi: 10.1186/1472-684X-12-7

45) Factors considered important at the end of life by patients, family, physicians, and other care providers. Steinhauser KE, Christakis NA, Clipp EC, McNeilly M, McIntyre L, Tulsky JA. JAMA. 2000 Nov 15;284(19):2476-82. doi: 10.1001/jama.284.19.2476.

46) In search of a good death: observations of patients, families, and providers. Steinhauser KE, Clipp EC, McNeilly M, Christakis NA, McIntyre LM, Tulsky JA. Ann Intern Med. 2000 May 16;132(10):825-32. doi: 10.7326/0003-4819-132-10-200005160-00011

47) The variability of critical care bed numbers in Europe. Rhodes A, Ferdinande P, Flaatten H, Guidet B, Metnitz PG, Moreno RP. Intensive Care Med. 2012 Oct;38(10):1647-53. doi: 10.1007/s00134-012-2627-8. Epub 2012 Jul 10.Intensive Care Med. 2012. PMID: 22777516

48) End-of-life care and circumstances of death in patients dying as a result of cancer in Belgium and the Netherlands: a retrospective comparative study. Meeussen K, Van den Block L, Echteld MA, Boffin N, Bilsen J, Van Casteren V, Abarshi E, Donker G, Onwuteaka-Philipsen B, Deliens L. J Clin Oncol. 2011 Nov 10;29(32):4327-34. doi: 10.1200/JCO.2011.34.9498. Epub 2011 Oct 11.

49) https://www.standaard.be/cnt/dmf20180606_03548418

50) https://www.hln.be/de-krant/beter-samenwerken~a889edb6/

51) Perceptions of the appropriateness of care in California adult intensive care units. Anstey MH, Adams JL, Mc Glynn EA. Crit Care. 2015 Feb 25;19(1):51. doi: 10.1186/s13054-015-0777-0.

52) Irrational Exuberance: Cardiopulmonary Resuscitation as Fetish. Rosoff PM, Schneiderman LJ. Am J Bioeth. 2017 Feb;17(2):26-34. doi: 10.1080/15265161.2016.1265163.

53) The Practice of Respect in the ICU. Brown SM, Azoulay E, Benoit D, Butler TP, Folcarelli P, Geller G, Rozenblum R, Sands K, Sokol-Hessner L, Talmor D, Turner K, Howell MD. Am J Respir Crit Care Med. 2018 Jun 1;197(11):1389-1395. doi: 10.1164/rccm.201708-1676CP.

54) Associations between end-of-life discussions, patient mental health, medical care near death, and caregiver bereavement adjustment. Wright AA, Zhang B, Ray A, Mack JW, Trice E, Balboni T, Mitchell SL, Jackson VA, Block SD, Maciejewski PK, Prigerson HG. JAMA. 2008 Oct 8;300(14):1665-73. doi: 10.1001/jama.300.14.1665.

55) ICU care associated with symptoms of depression and posttraumatic stress disorder among family members of patients who die in the ICU. Kross EK, Engelberg RA, Gries CJ, Nielsen EL, Zatzick D, Curtis JR. Chest. 2011 Apr;139(4):795-801. doi: 10.1378/chest.10-0652. Epub 2010 Sep 9.

56) Risk of post-traumatic stress symptoms in family members of intensive care unit patients. Azoulay E, Pochard F, Kentish-Barnes N, Chevret S, Aboab J, Adrie C, Annane D, Bleichner G, Bollaert PE, Darmon M, Fassier T, Galliot R, Garrouste-Orgeas M, Goulenok C, Goldgran-Toledano D, Hayon J, Jourdain M, Kaidomar M, Laplace C, Larché J, Liotier J, Papazian L, Poisson C, Reignier J, Saidi F, Schlemmer B; FAMIREA Study Group. Am J Respir Crit Care Med. 2005 May 1;171(9):987-94. doi: 10.1164/rccm.200409-1295OC. Epub 2005 Jan 21

57) Complicated grief after death of a relative in the intensive care unit. Kentish-Barnes N, Chaize M, Seegers V, Legriel S, Cariou A, Jaber S, Lefrant JY, Floccard B, Renault A, Vinatier I, Mathonnet A, Reuter D, Guisset O, Cohen-Solal Z, Cracco C, Seguin A, Durand-Gasselin J, Éon B, Thirion M, Rigaud JP, Philippon-Jouve B, Argaud L, Chouquer R, Adda M, Dedrie C, Georges H, Lebas E, Rolin N, Bollaert PE, Lecuyer L, Viquesnel G, Léone M, Chalumeau-Lemoine L, Garrouste M, Schlemmer B, Chevret S, Falissard B, Azoulay E. Eur Respir J. 2015 May;45(5):1341-52. doi: 10.1183/09031936.00160014. Epub 2015 Jan 22.

58) Understanding ethical climate, moral distress, and burnout: a novel tool and a conceptual framework. Dzeng E, Curtis JR. BMJ Qual Saf. 2018 Oct;27(10):766-770. doi: 10.1136/bmjqs-2018-007905.

59) Prevalence and factors of intensive care unit conflicts: the conflicus study. Azoulay E, Timsit JF, Sprung CL, Soares M, Rusinová K, Lafabrie A, Abizanda R, Svantesson M, Rubulotta F, Ricou B, Benoit D, Heyland D, Joynt G, Français A, Azeivedo-Maia P, Owczuk R, Benbenishty J, de Vita M, Valentin A, Ksomos A, Cohen S, Kompan L, Ho K, Abroug F, Kaarlola A, Gerlach H, Kyprianou T, Michalsen A, Chevret S, Schlemmer B; Conflicus Study Investigators and for the Ethics Section of the European Society of Intensive Care Medicine. Am J Respir Crit Care Med. 2009 Nov 1;180(9):853-60. doi: 10.1164/rccm.200810-1614OC. Epub 2009 Jul

60) Association of Physician Orders for Life-Sustaining Treatment With ICU Admission Among Patients Hospitalized Near the End of Life. Lee RY, Brumback LC, Sathitratanacheewin S, Lober WB, Modes ME, Lynch YT, Ambrose CI, Sibley J, Vranas KC, Sullivan DR, Engelberg RA, Curtis JR, Kross EK. JAMA. 2020 Mar 10;323(10):950-960. doi: 10.1001/jama.2019.22523.

61) Burnout syndrome among critical care healthcare workers. Embriaco N, Papazian L, Kentish-Barnes N, Pochard F, Azoulay E. Curr Opin Crit Care. 2007 Oct;13(5):482-8. doi: 10.1097/MCC.0b013e3282efd28a.

62) Burnout syndrome in critical care nursing staff. Poncet MC, Toullic P, Papazian L, Kentish-Barnes N, Timsit JF, Pochard F, Chevret S, Schlemmer B, Azoulay E. Am J Respir Crit Care Med. 2007 Apr 1;175(7):698-704. doi: 10.1164/rccm.200606-806OC

63) Non-beneficial therapy and emotional exhaustion in end-of-life care : Results of a survey among intensive care unit personnel. Hartog CS, Hoffmann F, Mikolajetz A, Schröder S, Michalsen A, Dey K, Riessen R, Jaschinski U, Weiss M, Ragaller M, Bercker S, Briegel J, Spies C, Schwarzkopf D; SepNet Critical Care Trials Group – Ethicus II Studiengruppe. Anaesthesist. 2018 Nov;67(11):850-858. doi: 10.1007/s00101-018-0485-7. Epub 2018 Sep 12.

64) Ethical climate and intention to leave among critical care clinicians: an observational study in 68 intensive care units across Europe and the United States.Van den Bulcke B, Metaxa V, Reyners AK, Rusinova K, Jensen HI, Malmgren J, Darmon M, Talmor D, Meert AP, Cancelliere L, Zubek L, Maia P, Michalsen A, Kompanje EJO, Vlerick P, Roels J, Vansteelandt S, Decruyenaere J, Azoulay E, Vanheule S, Piers R, Benoit D; DISPROPRICUS study group of the Ethics Section of the ESICM. Intensive Care Med. 2020 Jan;46(1):46-56. doi: 10.1007/s00134-019-05829-1

65) Financial hardship and the intensity of medical care received near death. Tucker-Seeley RD, Abel GA, Uno H, Prigerson H. Psychooncology. 2015 May;24(5):572-8. doi: 10.1002/pon.3624. Epub 2014 Jul 23.

66) Impact of aggressive management and palliative care on cancer costs in the final month of life. Cheung MC, Earle CC, Rangrej J, Ho TH, Liu N, Barbera L, Saskin R, Porter J, Seung SJ, Mittmann N. Cancer. 2015 Sep 15;121(18):3307-15. doi: 10.1002/cncr.29485. Epub 2015 May 29.

67) The frequency and cost of treatment perceived to be futile in critical care. Huynh TN, Kleerup EC, Wiley JF, Savitsky TD, Guse D, Garber BJ, Wenger NS. JAMA Intern Med. 2013 Nov 11;173(20):1887-94. doi: 10.1001/jamainternmed.2013.10261.JAMA Intern Med. 2013. PMID: 24018712

68) Critical care at the end of life: a population-level cohort study of cost and outcomes. Chaudhuri D, Tanuseputro P, Herritt B, D'Egidio G, Chalifoux M, Kyeremanteng K. Crit Care. 2017 May 31;21(1):124. doi: 10.1186/s13054-017-1711-4.

69) EQ-5D : a measure of health status from the EuroQol group. Rabin R, de Charro F. 2001 Ann Med 33:337-343

70) Satisfaction with quality of ICU care for patients and families: the euroQ2 project. Jensen HI, Gerritsen RT, Koopmans M, Downey L, Engelberg RA, Curtis JR, Spronk PE, Zijlstra JG, Ørding H. Crit Care. 2017 Sep 7;21(1):239. doi: 10.1186/s13054-017-1826-7.

71) Quality of dying and death in the ICU. The euroQ2 project. Gerritsen RT, Jensen HI, Koopmans M, Curtis JR, Downey L, Hofhuis JGM, Engelberg RA, Spronk PE, Zijlstra JG. J Crit Care. 2018 Apr;44:376-382. doi: 10.1016/j.jcrc.2017.12.015. Epub 2017 Dec 26.

72) The validation of visual analogue scales as ratio scale measurements for chronic and experimental pain. Price DD, McGrath PA, Rafii A, Buckingham B. Pain. 1983;14:45-56.

73) A validation study of the Hospital Anxiety and Depression Scale (HADS) in different groups of Dutch subjects. Spinhoven P, Ormel J, Sloekers PP, Kempen GI, Speckens AE, Van Hemert AM. Psychol Med. 1997 Mar;27(2):363-70. doi: 10.1017/s0033291796004382.

74) Quality of dying and death in the ICU. The euroQ2 project. Gerritsen RT, Jensen HI, Koopmans M, Curtis JR, Downey L, Hofhuis JGM, Engelberg RA, Spronk PE, Zijlstra JG. J Crit Care. 2018 Apr;44:376-382. doi: 10.1016/j.jcrc.2017.12.015. Epub 2017 Dec 26.

75) Potentially Inappropriate Treatments at the End of Life in Nursing Home Residents: Findings From the PACE Cross-Sectional Study in Six European Countries. Honinx E, Van den Block L, Piers R, Van Kuijk SMJ, Onwuteaka-Philipsen BD, Payne SA, Szczerbińska K, Gambassi GG, Finne-Soveri H, Deliens L, Smets T; PACE. J Pain Symptom Manage. 2020 Sep 8:S0885-3924(20)30728-4. doi: 10.1016/j.jpainsymman.2020.09.001.

76) Construct validation of the Dutch version of the impact of event scale. van der Ploeg E, Mooren TT, Kleber RJ, van der Velden PG, Brom D. Psychol Assess. 2004 Mar;16(1):16-26. doi: 10.1037/1040-3590.16.1.16.

15. Appendices

15.1. Appendix 1: Coaching protocol

The intervention that is central to this study consists of implementing coaching sessions with medical doctors. These sessions aim at facilitating self-reflective and empowering leadership, and at improving medical doctors' competence in managing team dynamics with regard to ethically sensitive medical topics, like hospitalized patients potentially receiving excessive care. Each time that at least two members of the team for which a doctor is responsible indicated that for one of the patients under treatment they were facing an ethical dilemma, the doctor is invited to have a coaching session. By stimulating open speech and reflection on ethically sensitive medical topics, like disagreements on interventions, difficulties in taking end-of-life decisions, or challenges of dealing with conflicting opinions in the doctor-patient relationship, the intervention intended at strengthening doctors' capacities to reflect and communicate about difficult decisions in uncertain circumstances and to guide their multidisciplinary teams in dealing with such topics.

Generally speaking, coaching is a form of "leadership development where a leader has a series of contracted and confidential conversations with a coaching psychologist or development expert. It is a form of organizational learning through one-to-one conversations, which facilitates development for an individual" (de Haan, Molyn & Nilsson, 2020, p. 2). The input coachees typically bring to coaching sessions can cover a spectrum of themes varying between personal dynamics influencing or disturbing work situations, dilemmas and difficulties related to their work setting or exploring opportunities for growth. During coaching "coachee and coach collaborate to assess and understand the coachee and his or her leadership developmental tasks, to challenge current constraints while exploring new possibilities, and to ensure accountability and support for reaching goals and sustaining development" (Ting & Hart, 2004, p. 116). It is supporting someone to adapt his/her relationship to a specific reality. Often an individual cannot overcome the issue by him-/herself as some variables are unknown and require multiple learning loops to understand specific internal and external dynamics which inhibit our performance (Heifetz, 1997). Since furthermore, "because of the personalized nature, the high confidentiality, and the possibility for deep understanding and challenge, coaching seems to work at relational and personal depths" (de Haan, Gray & Boneywell, 2019, p. 586). Consequently, we argued that coaching could support the aim of the study.

Study focus

In this study coaching is implemented with a clear focus: helping medical doctors to address ethically sensitive issues in multidisciplinary teams and to optimize their decision process and leadership for the team. In hospital teams doctors typically occupy two roles: the role of the single scientific expert and the role of the interacting leader. Stoller (Stoller, 2018) refers to a paradox as "the gap between the need for teamwork in healthcare and the doctor's traditional training as heroic lone healer. (….) And although teamwork matters immensely, doctors are neither selected nor trained to be team players" whilst it is under their responsibility that crucial decisions are taken and that team dynamics take shape. Our intervention aimed at supporting them in effectuating an empowering leadership style. As

Kets de Vries (Kets de Vries, 2014) indicates, “Empowering concerns the leader’s ability to delegate authority to others. An empowering leader involves others in the decision-making process thereby indicating his or her high expectations and confidence in them. An empowering leader also works to minimise secrecy and to create an open and transparent environment. He or she also tolerates mistakes and failures as part of the learning process.”

A key psychodynamic hypothesis guiding the intervention is that ethically sensitive medical topics are inherently anxiety provoking. They confront professionals with death and suffering, with uncertainty and powerlessness in aversive situations, and with the prospect that decisions and actions might have an aggravating impact on aversive situations. Such confrontations have a shocking effect and provoke mixed feelings. Shame, pity, compassion and guilt might come to the fore, but the bottom-line reaction to death and decay is desperate anxiety.

In her seminal psychodynamic studies on how medical professionals work, Menzies Lyth (1988) described that if this elementary anxiety is not faced and worked through, all kinds of dysfunctional defenses ruin collaborations and undermine thoughtful medical ethical decision-making. Working through means that challenging situations are faced and discussed in plain but respectful terms, such that the complexity of the situation is acknowledged and affective reactions are contained in the interactions between professionals, patients and families. Dysfunctional defensive reactions in their turn come to the fore as people avoid thoughtful deliberation about sensitive medical topics, and shy away from such situations, e.g. by avoiding close commitment to patients, fleeing in depersonalized and neutral interactions, focusing on technical details in treatments only, emotional unresponsiveness, or scheduling no time to address worries and concerns in team meetings.

Given this challenge, the coaching sessions aim at helping medical doctors to work through ethically sensitive medical topics and facilitate a culture of open speech in their teams, defined within the context of empowering leadership. We do so by helping them to: discern which sensitive themes were currently affecting their team; reflect on how the team is currently facing or avoiding difficult questions and discussions concerning these topics; evaluate their own role and strategies in facilitating or obstructing open reflection; reflect on how they and the team are in dealing with the emotional distress that ethically sensitive medical topics.
In 2018 Van den Bulcke and colleagues reviewed literature on medical ethical decision-making and based on psychometric research of a newly constructed questionnaire (EDMCQ) they discern seven key domains that make up a medical ethical decision-making climate: (1) self-reflective and empowering leadership by doctors; (2) practice and culture of open interdisciplinary reflection; (3) culture of not avoiding end-of-life decisions; (4) culture of mutual respect in the interdisciplinary team; (5) active involvement of nurses in end-of-life care and decision-making; (6) active decision-making by doctors; and (7) practice and culture of ethical awareness. In this study, we focus on coaching doctors in self-reflective and empowering leadership, which by itself will have an effect on all other factors in the team (see theoretical framework in the protocol).

Coaching method

The coaching we implemented started from the principles described by Kets de Vries (2006, 2007) and de Haan (2008, 2014, 2019). The sessions focused on discussing: (a) challenges and opportunities in dealing with ethical and medical dilemmas; (b) challenges and opportunities in relating with colleagues and team, and with patients and families; (c) challenges and opportunities in taking up a empowering leadership role. The coach aimed at increasing awareness and efficacy in dealing with these topics, and transferring these into leadership behavior, starting from a collaborative relationship with the coachee.

Kets de Vries' coaching methodology is based on the combination of two concepts: a psychodynamic perspective and a systemic perspective. This is defined as the 'clinical paradigm', a conceptual framework that builds on psychoanalytical concepts and techniques and which takes into account the dynamics of organizational behavior (Kets de Vries, 2006). He argues that leadership behavior is driven by the interplay between conscious and unconscious processes, the so-called 'inner theatre' – the roles we have developed over the course of life and which permanently influence our thinking. Therefore this coaching methodology intends to help the leader make sense of the invisible deeper thoughts, feelings, motives and anxieties that influence his/her cognitive processes such as decision-making and daily leadership behavior.
Next to intrapersonal dynamics, the leader is also influenced by interpersonal dynamics. A leader takes up distinct formal and informal roles in various social constructions that affect his/her intrapsychic life. This implies that decision-making behavior is undoubtedly influenced by the interpretation of these interactions. Finally, leaders also act according to organizational expectations, often derived from formal rules and so-called 'organizational myths', i.e. normative narratives that indicate how people should collaborate. As Kets de Vries (2006, p. 308) indicates, "subjection to these myths may come at the cost of personal responsibility and independence".

It is the primary task of the coach to help leaders to explore their unconscious and invisible psychodynamic processes and structures within the context of their organization's internal and social dynamics (Kets de Vries, 2007). "A well-trained developmental coach should at least be able to develop hypotheses and interventions both at an individual level as well as a group level" (Compernolle T., in Kets de Vries, M., Korotov, K., Florent-Treacy, E., 2007, p.35).

De Haan's coaching methodology is based on a relational model, in which the working alliance between coach and coachee is seen as a predictor of present and future leadership effectiveness (de Haan, Gray & Boneywell, 2019, p. 585). De Haan describes relational coaching as a dual active effort. On the one hand the coach is requested to understand all leadership dynamics from the perspective of the relationships the coachee is involved in, being previous, current and in-the-moment relationships. On the other hand the coach helps the coachee to make his/her professional relationships as strong and productive as possible – considered from the perspective of the coachee. Consequently, the coach needs "to explore regularly with the coachee how the relationship is progressing" (de Haan, 2008, p.53).

Research shows that "professional coaches who specialize in the coaching profession perceive more typical nondirective and client-centered coaching behaviors over time". It is argued that coaches "have a wide array of behavioral responses at their disposal and as they

mature, they will reflect on which of their interventions to use" (de Haan & Nilsson, 2017, p. 328). This might imply that starting from a client – centred approach requires the coach to apply a wider portfolio of potential responses and methods. By becoming more skillful over time, this will provide greater flexibility to design approaches specific to the individual (Cox, Bachkirova, Clutterbuck, 2010, p.419-420). However, this also means that the coach will always influence the conversation, both intentionally and unintentionally, by every contribution to that conversation. The coachee, as second element in the relationship, will equally bring a plethora of variables into the conversation. "An orderly, well-controlled conversation is out of the question; there are simply too many variables" (de Haan & Burger, 2014, p. 15). Consequently, de Haan argues it is the coach's responsibility to keep a window onto the coachee's contribution to the conversation because the coachee's issues are data and material to work with. And it is the reciprocal relation, where coach and coachee explore the map of experiences in the moment, that defines the foundation of the coaching work.

de Haan comes with an intervention model that gives the coach a framework to conduct the conversation, based on the assumption that the coach can make the intervention from two different perspectives: the direction of the contribution (exploring or suggesting) and the nature of the contribution (supporting or confronting). In the direction – perspective the coach can decide to rather lead vs. follow the coachee. In this perspective it is a matter of either constraining the coachee's thoughts, or following and liberating the coachee's thoughts. In the nature of the contribution – perspective the coach can choose between challenging and supporting the coachee. In this perspective it is a matter of either building on and reinforcing the coachee's strengths or bringing up the weaknesses and help overcoming them. (de Haan & Burger, 2014)

de Haan also contributes to the instrumentation of the coach by suggesting 4 distinct and interchangeable approaches. The first approach is person – focused, which is based on the counselling methods as developed by Carl Rogers. The coach is observing and supporting the coachee from the his/her perspective, "attempts to shift the coachee's attention inwards and is available primarily as an accepting and attentive listener" (de Haan, 2008, p. 15). The second approach is insight-focused, which is based on the tradition of psychodynamic coaching, as described by Kets de Vries. The coach is considering the coachee from an independent perspective and "attempts, with the coachee, to understand the issue from the inside" (de Haan, 2008, p. 15). The third approach is problem-focused, which is helping the coachee with an approach to solve a problem and consequently more directive in nature. "The coach attempts to improve the situation from the outside" (de Haan, 2008, p. 16). The fourth approach is solution-focused and is a particular form of directive coaching. The coach "attempts to convert problems into positive plans and solutions" and conducts a conversation "in which coach and coachee predominantly look to the future and consider times when the problem does not arise (de Haan, 2008, p. 16).

Summarised, de Haan argues in his model of relational coaching that there should be "no restriction on the specific interventions of the coach, either in terms of nature of the contributions or in terms of the order of contributions" (de Haan, 2008, p.54). This means the coach is holding the space for the coachee and can apply a variety of relational interventions depending on coachee's reality within the moment. Consequently, there are only 3 limiting conditions for the coach: the interventions should fit in with the framework's assumptions concerning learning, development and changes; they should be focused on supporting and

reinforcing the relationship and thus the coachee's learning and development process; and they should be in line with relevant ethical codes, which e.g. implies that the coach is sufficiently open to supervision (de Haan, 2008).

Coaching sessions

Each coaching session consists of an in-depth discussion of the decision making process around the case of the patient of which an alert was given.

The coach invites the coachee to describe and discuss:

- The currently challenging situation
- Perceived subjective reactions by him-/herself and others (patient, family, colleagues) to the situation
- Perceived challenges in the mutual communication about the situation, whereby attention is paid to characteristics of the own communication style and the style of the others involved
- Perceived challenges in the medical ethical decision process in the challenging situation, whereby attention is paid to differences and similarities in goals and values between all parties involved

By discussing these topics the coach aims at addressing and fostering insight in:

- Avoidance reactions in how the coached doctor, his team, and the patient and his family cope with the challenging situation
- Helping and obstructing relational patterns in relation to the team as well as in relation to the patient and his family
- Own communicative style
- Own decision making style
- Own strengths and weaknesses at the level of empowering leadership

Depending on the challenging situation and the coachee' style and trajectory the coach will direct sessions towards:

- Acknowledging and gaining insight in avoidance reactions, relational patterns, communicative processes, decision making processes and empowering leadership
- Confronting the coachee with processes he/she fails to acknowledge
- Reflecting with the coachee on dealing with and intervening upon such processes

Coaching intervention

Based on experiences from our expert panel, we implemented four coaching programs , each one spread over a period of 4 months. During a coaching program, we hypothesized that every doctor would receive between 8 and 16 coaching sessions of 1,5h each, which were conducted in 10 departments. During that period individual coaching took place in self-reflection and self-regulation with regard to ethical decision-making in patients potentially receiving excessive care and in coping with group dynamics in the interdisciplinary team, with the intention to achieve following specific objectives:

a) Learning to acknowledge the patient's (and relatives') subjective goals, emotions and values, and separate them from own and colleagues' subjective goals, emotions and values trigged by that situation

b) Learning to acknowledge patient's (and relatives'), colleagues' and own spontaneous defensive avoidance strategies in coping with difficult and aversive care-related situations, like end-of-life decisions.
c) Learning to identify and separate internal avoidance strategies from external barriers to better delignate the responsibilities of each stakeholder in the process
d) Learning to cope more effectively with these internal and avoidance strategies and external barriers to enable more appropriate and timely decisions for the benefit of the patient.
e) Learning to integrate newly acquired insights into an adapted way of thinking and relating with others to establish a sustainable effect with regards to ethical decision-making.
f) Learning to transfer these insights into empowering leadership behaviour which contributes to dialogue during the interdisciplinary meeting

Success indicators

1. The doctor indicated that the coaching intervention contributed to his/her ability on end-of-life decisions.
2. The doctor indicated he/she applies these skills in daily practice.
3. The doctor indicated that the coaching fostered job satisfaction.
4. The doctor indicated an increased collaboration with colleague staff.
5. The doctor indicated an increased collaboration with doctors in training.
6. The doctor indicated an increased collaboration with doctors from other disciplines.
7. The doctor indicated an increased collaboration with nursing colleagues.
8. The doctor deemed the coaching as valuable.
9. The doctor became more aware of his/her subjective reaction to the uncertainty of the prognosis.
10. The doctor became more aware of his/her subjective reaction to the anticipated decision.
11. The doctor described an increased insight on dealing with conflict.
12. The doctor demonstrated his/her ability to implement newly acquired insights during the interdisciplinary meeting.
13. The doctor applied empowering leadership skills during the interdisciplinary meeting.
14. The doctor gave proof through specific comments of insight into the perspective of the patient.
15. The doctor gave proof through specific comments of insight into the perspective of the family.
16. The doctor gave proof through specific comments of insight into the perspective of team members.
17. The doctor gave proof through specific comments of insight into the perspective of the referring physician.
18. The doctor gave proof through specific comments being able to separate internal avoidance strategies from external barriers.
19. The doctor was able to mention barriers in ethical decision making for specific cases.
20. The intervention took place in an appropriate coaching setting (i.e. confidential, discrete, serene, not be pressured by time,…).

Coaching conversation

Whilst each coaching conversation is a dynamic interaction between coach and coachee, the workflow of the coaching conversation was standardised. As referred, multiple learning loops within the conversation might be needed to understand specific internal and external dynamics which inhibit our performance (Heifetz, 1997). In order to give doctors the possibility to achieve this objective, each coaching conversation therefore followed a specific workflow with multiple learning loops to foster the reflection process within the moment (Killburg, 2000).

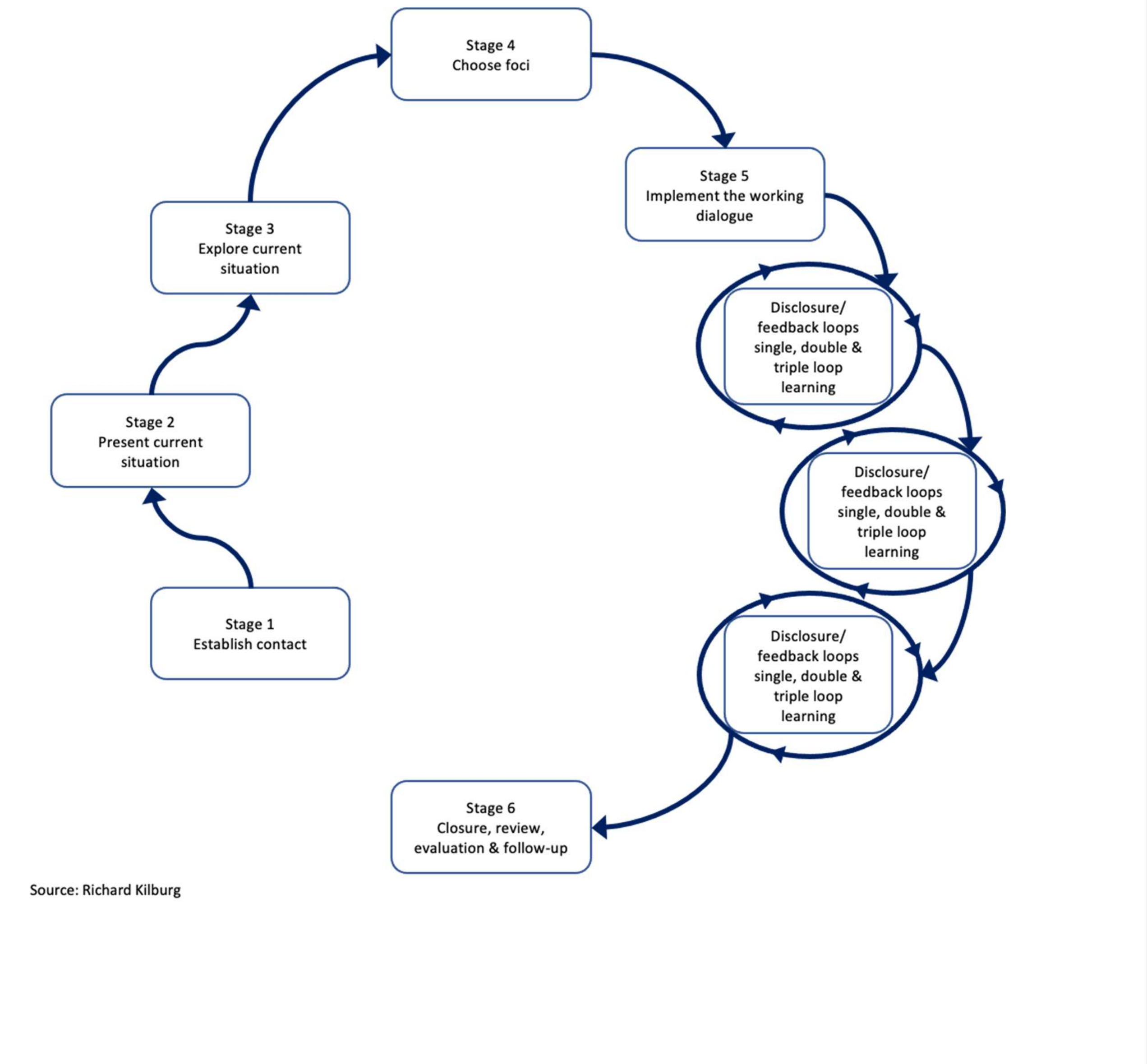

Quality assurance

To secure the quality of the intervention the coach worked under the guidelines of the Ashridge Code of Conduct (see appendix).

The quality of the coaching we implemented was also supervised by an independed third-party supervisor; governed by ethical standards and principles as adopted from de Haan (de

Haan, 2019). Five moral foundations which support integrity in coaching and research were applied: independence to guard the doctor's autonomy, informed consent so the doctor knows what he/she is getting involved in, confidentiality to safeguard the doctor, respect and diversity to allow multiple voices and perspectives and care about vulnerable parties, integrity and trust to comply with legislation and to handle data in an ethical way.

This supervisor had is a normative, formative and restoring role (Proctor, 2006). The normative role of the supervision aimed at monitoring the quality of the coaching methodology and ethical aspects with regard to the study aims. The formative role aimed at supporting the coach in further developing and refining his/her skills in general and more specifically with regard to the study aims. The restorative role aimed at guarantying the energy of the coach and at resolving potential conflicts due to emotional or unconscious dynamics in the team.

Coaching Supervision is about ensuring high quality coaching provision and takes the form of ongoing meetings between the supervisor and the coach. One way of looking at the process of supervision is provided by the Seven-eyed model (Hawkins and Shohet, 2000). Originally developed for use with psychotherapists and counsellors, it is now being applied to coaching and mentoring. It describes the 7 areas that supervision can focus on:

1. **The doctor**: The focus is on the doctor's situation – the case the doctor wants help with, how they present the issues and the choices they are making.
2. **The coach's Interventions**: The focus is on the interventions the coach made, how and why they made them, and what else they might have done.
3. **The relationship between the coach and doctor**: The focus is on neither the coach nor the doctor but on the conscious and unconscious interactions between the two of them, so that the coach develops a better understanding of the dynamics of the coaching relationship.
4. **The coach**: The focus is on the coach's own experience as an instrument for registering what is happening beneath the surface of the doctor's system.
5. **The supervisory relationship**: The focus is on what the coach has absorbed from the doctor's system and how it may be playing out in the relationship between coach and supervisor.
6. **The supervisor's self-reflection**: The focus is the supervisor's "here and now" experience with the coach and how this can be used to shed light on the coach/ doctor relationship.
7. **The wider context**: The focus is on the wider organisational, social, cultural, ethical, and contractual context within which the supervision is taking place.

In focusing on areas 1-3, the supervision is concerned with reflecting on the coaching session itself – its content, the interventions made, and the dynamics of the coaching relationship.

In areas 4-6, the supervision is concerned with the coaching session as it is reflected in the here and now experience of the supervision session.

The value of this model is that it maps the areas that supervision can focus on, making it easier to ensure that we have covered the ground.

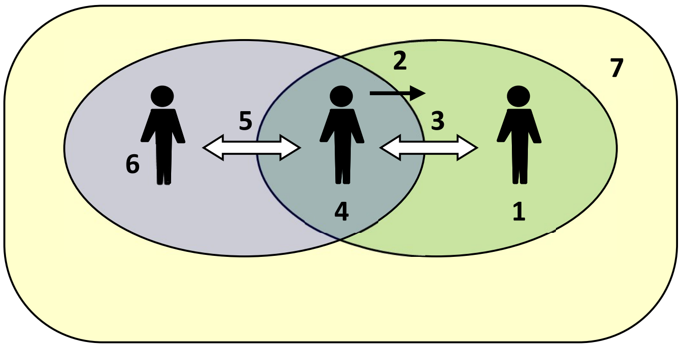
2
7
5
3
6
4
1

Bibliography

Cox, E., Bachkirova, T., Clutterbuck, D., (2010). *Handbook of coaching.* London: Sage

de Haan, E. (2008). *Relational Coaching*. West Sussex: Wiley

de Haan, E. Burger, Y., (2014). *Coaching with colleagues.* Basingstoke: Palgrave MacMillan

de Haan, E. (2019). *Critical moments in executive coaching.* New York: Routledge

de Haan, E., Gray, D.E. & Bonneywell, S. (2019). Executive coaching outcome research in a field setting: A near-randomized controlled trial study in a global healthcare corporation. Academy of Management Learning and Education, 18.4, 1- 25. https://doi.org/10.5465/amle.2018.01588

de Haan, Large-scale survey of trust and safety in coaching supervision: Some evidence that we are doing it right. *International Coaching Psychology Review* l *Vol. 12 No. 1 March 2017*

de Haan,E., Nilsson, V. O. (2017). Evaluating coaching behavior in managers, consultants, and coaches: a model, questionnaire and initial findings. Consulting Psychology Journal: Practice and Research, 69.4, 315-333.

de Haan, E., Molyn, J., & Nilsson, V. O. (2020). New findings on the effectiveness of the coaching relationship: Time to think differently about active ingredients? *Consulting Psychology Journal: Practice and Research.* Advance online publication. https://doi.org/10.1037/cpb0000175

Hawkins, P., Shohet, R. (2012). *Supervision in the helping professions.* Maidenhead: McGraw-Hill Education

Kets de Vries, M. (2006). *The leader on the couch.* West Sussex: Wiley

Kets de Vries, M., Korotov, K., Florent-Treacy, E. (2007). *Coach and couch.* New York: Palgrave Macmillan

Kets de Vries, M. (2014). *Global Executive Leadership Mirror; facilitator guide*. London: KDVI

Kilburg, R. (2000). *Executive Coaching*. Washington: American Psychological Association

Proctor, B. (2006). Contracting in supervision. In C. Sills (Ed.). *Contracts in counselling &*

psychotherapy (pp. 161-174). London: Sage Publications Ltd.

Stoller, J.K. (2018). Developing Physician Leaders
A Perspective on Rationale, Current Experience, and Needs

Ting, S., Hart; E.W. (2004). Formal coaching. In: C.D. McCauley, E. Van Velsor (Eds.), The Center for Creative Leadership Handbook of Leadership Development, John Wiley & Sons, San Francisco (2004), pp. 116-150

Van den Bulcke et al. (2018). Ethical decision-making climate in the ICU: theoretical framework and validation of a self-assessment tool. *BMJ Qual Saf, 27(10),*781–789
